# Kinetics of Calcite Nucleation onto Sulfated Chitosan Derivatives and Implications for Water–Polysaccharide Interactions during Crystallization of Sparingly Soluble Salts

**DOI:** 10.1021/acs.cgd.4c00602

**Published:** 2024-07-11

**Authors:** Brenna M. Knight, Ronnie Mondal, Nizhou Han, Nicholas F. Pietra, Brady A. Hall, Kevin J. Edgar, Valerie Vaissier Welborn, Louis A. Madsen, James J. De Yoreo, Patricia M. Dove

**Affiliations:** † Department of Chemistry, Virginia Tech, Blacksburg, Virginia 24061, United States; ‡ Department of Geosciences, Virginia Tech, Blacksburg, Virginia 24061, United States; § Macromolecules Innovation Institute, Virginia Tech, Blacksburg, Virginia 24061, United States; ∥ GlycoMIP, Virginia Tech, Blacksburg, Virginia 24061, United States; ⊥ Department of Sustainable Biomaterials, Virginia Tech, Blacksburg, Virginia 24061, United States; # Physical Sciences Division, Physical and Computational Sciences Directorate, Pacific Northwest National Laboratory, Richland, Washington 99352, United States; ∇ Department of Materials Science and Engineering, University of Washington, Seattle, Washington 98195, United States; ○ Department of Materials Science and Engineering, Virginia Tech, Blacksburg, Virginia 24061, United States

## Abstract

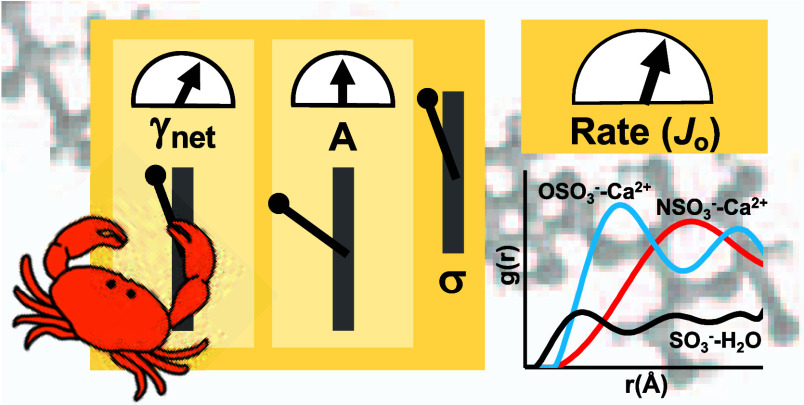

Anionic macromolecules are found at sites of CaCO_3_ biomineralization in diverse organisms, but their roles in crystallization are not well-understood. We prepared a series of sulfated chitosan derivatives with varied positions and degrees of sulfation, DS(SO_3_^–^), and measured calcite nucleation rate onto these materials. Fitting the classical nucleation theory model to the kinetic data reveals the interfacial free energy of the calcite–polysaccharide–solution system, γ_net_, is lowest for nonsulfated controls and increases with DS(SO_3_^–^). The kinetic prefactor also increases with DS(SO_3_^–^). Simulations of Ca^2+^–H_2_O–chitosan systems show greater water structuring around sulfate groups compared to uncharged substituents, independent of sulfate location. Ca^2+^–SO_3_^–^ interactions are solvent-separated by distances that are inversely correlated with DS(SO_3_^–^) of the polysaccharide. The simulations also predict SO_3_^–^ and NH_3_^+^ groups affect the solvation waters and HCO_3_^–^ ions associated with Ca^2+^. Integrating the experimental and computational evidence suggests sulfate groups influence nucleation by increasing the difficulty of displacing near-surface water, thereby increasing γ_net_. By correlating γ_net_ and net charge per monosaccharide for diverse polysaccharides, we suggest the solvent-separated interactions of functional groups with Ca^2+^ influence thermodynamic and kinetic components to crystallization by similar solvent-dominated processes. The findings reiterate the importance of establishing water structure and properties at macromolecule–solution interfaces.

## Abstract 2

This study measured the kinetics of CaCO_3_ nucleation onto a series of sulfated chitosan derivatives as macromolecular models for polysaccharides in the organic matrix. The thermodynamic (γ_net_) and kinetic (*A*) contributions to the rate correlate with degree of macromolecule sulfation. Molecular dynamics simulations show functional group type, density, and position modulate water structuring and Ca^2+^ interactions at the polysaccharide−solution interface.

## 1 Introduction

Many organisms use an organic matrix (OM) of macromolecules to form mineralized structures that serve diverse functions including skeletal support, buoyancy, and light harvesting.^1−9^ Charged biopolymers are of particular interest, with carboxylated proteins receiving extensive attention in the biomineralization literature.^2,10−21^ Sites of calcium carbonate (CaCO_3_) biocrystallization are also rich in polysaccharides and proteoglycans that are heavily functionalized with sulfate (SO_3_^–^), carboxyl (COO^–^), and phosphate (PO_3_^–^) groups.^3,22−26^ From cnidarians to chordates and algae (e.g., Table 1), glycomaterials with complex configurations of charged functional groups are found at sites of CaCO_3_ mineralization.

**Table 1:** Diverse Organisms Contain Sulfated Polysaccharides (PS) at Sites of CaCO_3_ Mineralization

	organism	association	ref
cnidarians	Acropora millepora*	sulfated arabinan	(52)
*stony corals	Stylophora pistillata*	sulfated arabinan	
	Astroides calycularis*	sulfated glucuronic acid	(53)
	Balaophyllia europaea*	sulfated glucuronic acid	
arthropods	*Ucides cordatus* (land crab)	sulfated glycoproteins	(54)
	lobster	sulfated glycoproteins	(55)
	crayfish	dermatan-, keratan-, and chondroitin-sulfate GAGs^a^	(56 and 57)
annelids	*Hydroides dianthus*	sulfated PS	(58)
mollusks	*Mercenaria mercenaria*	sulfated GAG^a^	(59 and 60)
	*A. pleuronectus*	heparan sulfate, other GAGs^a^	(61)
	*A. cynea*	glycosaminoglycans	(62)
	*Tapes phylippinarum*	heparin, 81% trisulfated disaccharide^b^	(63)
	*C. kingii*	sulfated GAG^a^	(64)
	*C. disrupta*	sulfated GAG^a^	
	*A. brasiliana*	heparin	(65 and 66)
	*A. brasiliana*	chondroitin sulfate	(65)
	*T. gibbus*	heparan sulfate and chondroitin sulfate	
	*Pomacea* sp.	heparan sulfate and chondroitin sulfate	
echinoderms	Neocrinus decorus*	sulfated PS	(67)
*crinoids	*Endoxocrinus parrae* parrae*	sulfated PS	
	Metacrinus rotundus*	sulfated PS	
	Hypalocrinus naresianus*	sulfated PS	
	*Ludwigothurea grisea* (sea cucumber)	fucose-linked chondroitin sulfate	(68 and 69)
	*Arbacia lixula* (sea urchin)	sulfated PS, sulfated proteins	(70)
chordates	*Platycephalus bassensis* (flathead fish)	sulfate, assumed proteoglycans	(71)
	mouse otoconia	serine rich protein with chondroitin sulfate linkages	(72)
	*S. plicata* (sea squirt) tunic	sulfated glycans	(73)
	*G. gallus* (emu) egg	sulfated proteins, keratan sulfate	(74)
	*D. novahollandiae* (crocodile) egg	sulfated proteins, keratan sulfate	
	*C. moreletii* (chicken) egg	sulfated proteins, keratan sulfate	
	*Hadrosauridae* (dinosaur) egg	sulfated proteins, keratan sulfate	
	collagen systems	GAGs^a^ + proteoglycans	(75)
algae	*Heterostegina depressa*	sulfated GAG^a^	(76)
*red algae	*Calliarthron cheilosporioides*	sulfated galactans, carrageenan, and agaran	(77)
**coccolithophores	Coralliina piluifera*	sulfated xylogalactan, “aragan sulfate-like” glucan	(78 and 79)
	Joculator maximus*	sulfated xylogalactan	(80)
	Emiliania huxleti**	sulfated mannan	(81−83)
	Pleurochrysis carterae**	sulfated galacturonan	(81 and 84)

a GAG = glycosaminoglycan.

b This heparin is more sulfated than bovine and porcine-associated heparins.

In marine animals, sulfate-functionalized macromolecules are often present alongside chitin, an abundant, structural, amide-functionalized polysaccharide.^27−29^ For example, the internal shell of the cuttlefish (i.e., cuttlebone) has two main components: a chambered body part and a dorsal shield.^30,31^ Chitin fibers encase CaCO_3_ in both parts of the cuttlebone, but other macromolecules (many sulfated) are found at specific sites and contain unique chitin-binding domains. These macromolecules are proposed to direct CaCO_3_ formation into the pillars and lamellae that produce chambers or terraces that, in turn, form compact sublayers of the shield.^32−34^ Similar associations are described in other organisms, including mollusks,^35,36^ brachiopods,^35^ lobsters,^37^ barnacles,^24^ coralline algae,^38^ and coccolithophores.^39,40^

Understanding how the composition of macromolecules leads to complex natural composites also holds promise for environmental, clean energy, and biomedical applications.^41−43^ For example, tailored dispersions of CaCO_3_ could enhance the capability of biopolymers to remove heavy metals and pollutants for water treatment and soil remediation.^44−46^ By tuning polysaccharide functionality for biomedical applications, CaCO_3_ nucleation can be controlled to encapsulate drugs or to regulate timing for optimal release in the digestive system.^20,47,48^ Functionalized polysaccharides could further combine the traditional concepts of neutral template (chitin) and promoter (anionic macromolecules), giving rise to unique mineral-macromolecule materials for tissue scaffolding of other sparingly soluble salts such as calcium phosphates.^49−51^

Disparate experimental methods have been used to investigate the effect of sulfated macromolecules on CaCO_3_ nucleation (Table S1). Several studies note that SO_3_^–^ groups, associated with glycomaterials or proteins, may have energetic, kinetic, and stereochemical effects on CaCO_3_ mineralization.^2,13−16,85−93^ However, most investigators used qualitative approaches and produced conflicting reports of the influence of sulfated macromolecules upon mineralization. While these descriptions of impacts on crystal polymorph and/or morphology provide insight, such approaches do not provide significant mechanistic or quantitative information. Further, most studies probed biomolecule effects in aqueous suspensions and rarely investigated chitinous materials or substrates. For example, aqueous polystyrene sulfonate (PSS), which contains a SO_3_^–^ group on each repeat unit, promoted the formation of amorphous calcium carbonate (ACC)^94^ and vaterite^95−97^ in solution, often by forming aggregates of particles via nonclassical pathways.^98,99^ However, in the presence of progressively more sulfonated polystyrene films, the calcite polymorph was formed, and the number of crystallites increased with sulfonate density.^100^

Classical nucleation theory (CNT) provides a theoretical framework for building comprehensive models of how macromolecule composition and functional group organization control mineralization. The free energy barrier to crystal nucleation (Δ*g*_c_) can provide a thermodynamic basis for establishing how functional groups, as macromolecules or surface-assembled monolayers, regulate crystallization.^91,93,101−105^ Δ*g*_c_ is dependent on the cube of the interfacial free energy of the CaCO_3_–polysaccharide–solution system (γ_net_) such that small surface energy changes profoundly impact nucleation rates (e.g., Section 3.1). Calcite is an exemplary CaCO_3_ polymorph for experimental investigations of nucleation energetics. An understanding of the effect of macromolecule composition on the nucleation of this sparingly soluble salt can also provide insight into the biologically controlled formation of other low-solubility materials, including phosphates, sulfates, and some oxides/hydroxides.

A previous study demonstrated how CNT can be used to understand inorganic crystallization onto macromolecules by quantifying the kinetics of calcite nucleation for three notable polysaccharides: chitosan, alginate, and heparin.^91^ The interfacial free energy barrier to forming calcite on these materials was linearly correlated with net charge of the polysaccharide. Heparin (sulfated and carboxylated) and alginate (carboxylated) presented the highest energy barriers to nucleation (74 and 75 mJ m^–2^, respectively) compared to near-neutral (at pH 10.8) chitosan (51 mJ m^–2^). The relationship shows the activity of sulfate groups can equal that of carboxylates in regulating mineralization. To our knowledge, however, little other mechanistic understanding has been reported about how sulfate groups, or other features such as functional group position, conformation, or molecular weight, influence mineralization rates.

Chitosan provides an excellent model material for establishing how functional groups influence the energy barrier to crystallization. In addition to its structural similarities to chitin and the glycosaminoglycans that have been associated with biomineralization, chitosan offers additional advantages including its relatively simple composition and its solubility in mildly acidic solution, making it far easier to process than refractory chitin. These characteristics create the opportunity to tune structure–function relationships by systematically and selectively introducing chemical functional groups. We show that by derivatizing chitosan into a series of well-characterized compositions for systematic studies of mineralization, a quantitative and broader framework for macromolecular controls on mineralization can be established.

**Figure 1:**
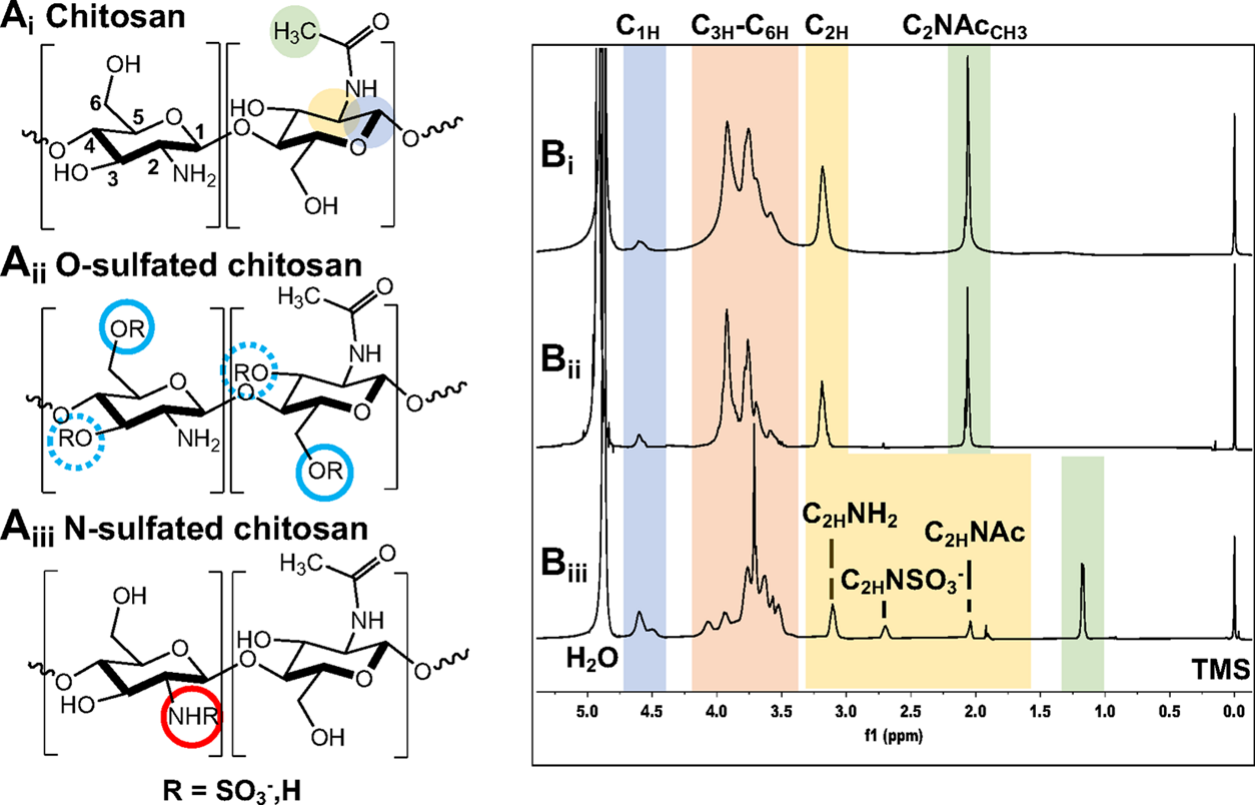
Natural chitin is deacetylated by alkaline hydrolysis to yield (A_i_) chitosan (control). This material was derivatized by two methods to prepare (A_ii_) *O*-sulfated chitosan (where most sulfate groups (R = SO_3_^–^) are on the C_6_O-position (solid circle) but can also be present at the C_3_O-position (dashed)) and (A_iii_) *N*-sulfated chitosan. ^1^H NMR spectra of (B_i_) chitosan, (B_ii_) *O*-sulfated chitosan, and (B_iii_) *N*-sulfated chitosan show the characteristic proton peaks used to quantify DS(Ac).

In this three-part study, we combine polymer chemistry and crystal growth science to test the hypothesis that sulfate density and position regulate the kinetics of CaCO_3_ nucleation onto chitosan through systematic controls on Ca^2+^–H_2_O–polysaccharide interactions. We first synthesize a series of chitosan (Figure 1A_i_) derivatives with variable positions (C_6_O- or C_2_N- (Figure 1A_ii,iii_)) and degrees of substitution of sulfate (DS(SO_3_^–^) = 0.1–0.8). Using these materials, we then measure the rate of calcite nucleation for a series of constant chemical driving force (supersaturation) conditions. By evaluating the rate data through the lens of CNT, we quantify relationships between sulfate density, position, and interfacial free energy of CaCO_3_ nucleation. In parallel, we perform molecular dynamics (MD) simulations to examine Ca^2+^–H_2_O–sulfated chitosan interactions and to better understand how sulfate density influences water and Ca^2+^ organization at the polysaccharide–solution interface.

## 2 Experimental Section

Unless otherwise noted, all materials were purchased from Millipore Sigma and used as received without further purification.

### 2.1 Synthesis of Sulfated Chitosan Derivatives

Seven chitosan materials were synthesized and purified for the experimental measurements then characterized by multiple approaches (Table 2). Sulfate groups (−SO_3_^–^) were introduced to the *O*- or *N*-positions yielding chitosan materials with sulfate or sulfonate groups, respectively. For simplicity, we refer to both materials as sulfated.

**Table 2:** Chemical and Physical Properties of the Chitosan Materials Investigated in This Study

notation for materials^a^	DS(SO_3_^–^)^b^	DS(Ac)^c^	DS(NH_2_) no XL^d^^,^^e^	max % XL^d^^,^^f^	DS(NH_2_) max XL^d^^,^^g^	diffusion coeff.(×10^–11^ m_2_ s^–1^)^h^	est. MW^i^ (kDa)
chitosan A^j^	0	0.24	0.76	43	0.33	0.31	370
chitosan B^k^	0	0.10	0.90	0		0.17	295
OSC 0.23	0.23	0.26	0.74	45	0.33	6.2	29
OSC 0.42	0.42	0.22	0.78	47	0.37	7.5	23
OSC 0.77	0.77	0.22	0.66	49	0.32	4.4	47
NSC 0.13	0.13	0.12	0.75	43	0.32	6.4	28
NSC 0.28	0.28	0.24	0.48	44	0.21	9.7	12
NSC 0.47	0.47	0.14	0.41	45	0.18	4.1	37

a The OSC and NSC notation denotes sulfation that is primarily at the *O*- or *N*-position, respectively, (e.g., Figure 1A_ii,iii_). The number that follows gives DS(SO_3_^–^) of the material.

b Determined by elemental analysis.

c Determined by ^1^H NMR.

d XL = cross-linking (Section 2.4.1).

e Determined by difference (1 – DS(Ac) – DS(NSO_3_^–^)).

f Determined by eq 7.

g Determined by multiplying DS(NH_2_) no XL by Max % XL.

h Determined by NMR diffusometry.

i MW = molecular weight.

j Control. Cross-linked during sample preparation (Section 2.4.1).

k Control. Not cross-linked during sample preparation (Section 2.4.1).

#### 2.1.1 *O*-Sulfation of Chitosan

Following the method for homogeneous sulfation of chitosan reported by Zhang et al.,^106^ medium molecular weight (MW; see Section 2.3.3 below) chitosan (0.50–1.02 g, degree of acetylation (DS(Ac)) = 0.24 by ^1^H NMR) was dissolved in formic acid (15–20 mL) at ambient temperature. *N*,*N*-Dimethylformamide (DMF, 95–200 mL, Thermo Fisher Scientific) was added, and the solution was stirred for 2 h. Chlorosulfonic acid (HSO_3_Cl, 2–7 mL) in 50 mL DMF was added dropwise over 30 min and solution temperature raised to 50 °C for 3 h. Once cooled to ambient temperature, the reaction solution was added to a saturated solution of NaOAc in ethanol (EtOH) to obtain a precipitate that was subsequently washed with EtOH/H_2_O (8/2, v/v) then redissolved in deionized (DI) water. The resulting solution was neutralized with NaOH, dialyzed (Thermo Fisher Scientific, MW cutoff 3.5 kDa) against water, and freeze-dried. Table S2 provides experimental parameters for each *O*-sulfated chitosan (OSC, Figure 1A_ii_).

#### 2.1.2 *N*-Sulfation of Chitosan

To prepare *N*-sulfated chitosan (NSC, Figure 1A_iii_), the methods of Holme and Perlin^107^ were followed with few modifications. Briefly, chitosan (0.35–0.51 g, DS(Ac) = 0.24) was dispersed in DI water (50–150 mL) and stirred overnight at 40 °C. Na_2_CO_3_ (0.45–1.20 g) and Me_3_N–SO_3_ (0.88–2.05 g, Thermo Fisher Scientific) were added to the reaction mixture and stirred for a minimum of 4 h. Experimental details for the preparation of each *N*-sulfated material are given in Table S2. At end of the reaction, solutions were successively dialyzed against DI water, followed by DI water containing Amberlite resin IR120 H^+^ form (Honeywell Fluka, washed with DI H_2_O before use), 0.025 M NaOH, and water again. Final products were then freeze-dried.

### 2.2 Carboxymethyl Chitosan (CMCS)

To test whether the impact of negatively charged groups on CaCO_3_ nucleation can be generalized to include carboxyl moieties, we performed nucleation experiments carboxymethyl chitosan (CMCS, see Section 3.5, DS(O–COO^–^) = 1.3, DS(N–COO^–^) = 0.2, DS(Ac) = 0.24; Zhou et al.).^108^ For the nucleation experiments, the CMCS was electrodeposited onto gold-coated mica using established methods.^91,109−112^

### 2.3 Characterization of Sulfated Chitosan Materials

#### 2.3.1 ^1^H NMR

To determine DS(Ac) of all chitosan materials and DS(SO_3_^–^) of *N*-sulfated materials, ^1^H spectra were acquired on a Bruker Avance II spectrometer at 500 MHz (120 scans, 2 s relaxation delay). All samples were analyzed as solutions (∼2% w/v) in D_2_O containing 0.05 wt % tetramethylsilane (TMS) as internal standard at 25 °C.

The chitosan spectra (Figure 1B_i_) were consistent with others reported in the literature.^106,113,114^ The acetyl methyl proton resonance was at 2.06 ppm, and C_2_ protons of acetylated and deacetylated monosaccharides appeared as one peak at 3.19 ppm. Resonances between 3.50 and 4.10 ppm were attributed to the C_3_–C_6_ carbon backbone protons. The anomeric C_1_ protons of acetylated and deacetylated monosaccharides appeared to be present at 4.60 ppm as one resonance, however two resonances may exist, with one being hidden by the H_2_O signal. For the primarily *O*-sulfated chitosan materials, the spectra (e.g., OSC 0.42, Figure 1B_ii_) were indistinguishable from the chitosan spectra.

Spectra of *N*-sulfated chitosan materials (e.g., NSC 0.47, Figure 1B_iii_) showed a shift of the methyl protons to 1.18 ppm, and the C_2_ proton peaks were resolved between monosaccharides, consistent with Holme and Perlin.^107^ The acetylated monosaccharides (C_2_**H**NHAc) resonated at 2.05 ppm, the resonance for *N*-sulfated monosaccharides (C_2_**H**NHSO_3_^–^) was at 2.70 ppm, and that for deacetylated (and therefore aminated) monosaccharides (C_2_**H**NH_2_) was at 3.11 ppm. C_3_–C_6_ protons were between 3.30 and 4.25 ppm. We also observed two C_1_–**H** resonances at 4.50 and 4.60 ppm. As with chitosan, another may be hidden by the H_2_O resonance. One of the *O*-sulfated materials exhibited partial *N*-sulfation (and therefore three C_2_ resonances). For clarity, we refer to this material as *O*-sulfated (DS(OSO_3_^–^) 0.65, DS(NSO_3_^–^) = 0.12).

The DS(Ac) of each chitosan material was determined using the integrals (I) of the peaks for the acetyl group methyl protons and backbone protons (C_1_–C_6_) by the equation:
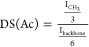
1

Using the C_2_–**H** peak integrals, the DS(SO_3_^–^) of the *N*-sulfated materials was determined:
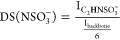
2

#### 2.3.2 Elemental Analysis

Elemental analysis via combustion was performed by Midwest Micro Lab in triplicate on all materials to determine the proportions of carbon, nitrogen, hydrogen, and sulfur. Assuming that all of the detected sulfur could be attributed to attached sulfate groups, the DS(SO_3_^–^) of materials was determined by the relation:
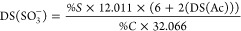
3

Estimates of DS(SO_3_^–^) for *N*-sulfated materials obtained by ^1^H NMR and elemental analysis methods were in good agreement (difference in DS ≤ 0.01).

#### 2.3.3 Estimates of Molecular Weight

Molecular weight determination of chitosan is difficult; this is often true of charged polysaccharides (e.g., Kasaai et al.).^115^ In this study, we utilized three approaches to estimate the molecular weight/degree of polymerization (DP) of chitosan and the derivatives we prepared.

##### 2.3.3.1 Viscometry

Traditionally, the viscosity average molecular weight (*M*_v_) of chitosan has been determined using the Mark–Houwink equation:^116^
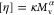
4where [η] is the intrinsic viscosity, determined by measuring the viscosity of a series of solutions of dilute concentrations, and κ and α are constants for a given polymer-solvent-temperature system related to polymer solubility and stiffness. Values of κ and α are calculated using a series of monodisperse samples and are tabulated for a large range of (primarily synthetic) polymers.^117,118^ Kasaai et al.^115^ developed expressions to estimate κ and α for chitosan based on DS(Ac), pH, and ionic strength.

Using a Brookfield D_v2_T viscometer with a SC4–18(18) spindle rotating at 200 rpm, we measured the viscosity of dilute aqueous solutions of the chitosan starting material (1–5 mM) and determined an intrinsic viscosity (458 mL g^–1^, Figure S1). The Kasaai et al.^115^ equations were used to determine κ and α (resulting in values of 4.44 × 10^–5^ mL g^–1^ and 1.26, respectively, DS(Ac) = 0.24, pH = 4.5, ionic strength = 0.01 M). By this approach, using eq 4, we estimated the starting chitosan material (prior to derivatizing) had *M*_v_ = 370 kDa which is greater than the wide manufacturer-provided range of 190–310 kDa. Previous studies have used reported values of κ and α for heparin to determine *M*_v_ of sulfated chitosan derivatives.^119−121^ However, heparin structure can vary greatly between samples, and its behavior in solution is unlike that of chitosan. These limitations, compounded with the time and material intensive nature of determining intrinsic viscosity (and/or further determining the κ and α values), led us to conclude that viscometry was not the ideal method for estimating MW of the sulfated chitosan derivatives.

##### 2.3.3.2 Aqueous Size Exclusion Chromatography (aqSEC)

To estimate the weight-average molecular weight (*M*_w_) of the sulfated chitosan derivatives, aqSEC was performed using instrumentation consisting of Wyatt Technologies TRIOS II light scattering and Optilab T-REX refractive index detectors. Dextran standards and a d*n*/d*c* of 0.1380 mL g^–1^ were used for calibration. Each material (5 mg) was dissolved into 1.5 mL of pH 3.0 DI H_2_O/acetic acid (mobile phase). Samples were eluted using a Shodex OHpal LB-806 M column (50 °C) with a Shimadzu LC-20AD pump flowing at 1.0 mL min^–1^. Unfortunately, only two materials eluted properly: one *O*-sulfated material (OSC 0.77) and one *N*-sulfated material (NSC 0.47) to obtain *M*_w_ estimates of 46 and 37 kDa, respectively (Figure S2)**.** The significantly lower *M*_w_ of these materials compared to the *M*_v_ of the starting material is unsurprising given that the chitosan derivatization to sulfated products required high temperatures and highly acidic conditions, likely causing some hydrolysis of anomeric linkages.

##### 2.3.3.3 Diffusion Coefficient (NMR Diffusometry)

An alternate version of the Mark–Houwink equation relates *M*_v_ and diffusion coefficient (*D*):
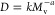
5where *k* and *a* are constants for a given polymer-solvent-temperature system but differ from κ and α used for viscometry methods (values of *k* and *a* are also tabulated for many synthetic polymers). While the same issue of not having *k* and *a* values for the sulfated chitosan materials persists, the diffusion coefficients of all materials can be rapidly measured and compared by NMR diffusometry.

Diffusion coefficients for all materials were determined (Table 2) using the pulsed-gradient stimulated echo sequence (PGSTE) run on a 9.4 T (T) Bruker Avance III wide-bore spectrometer at 25 °C with a Diff50 gradient coil. During an experiment, the intensity (*I*) of the signal decreases with increasing gradient strength, *g*, according to the Stejskal–Tanner relationship:^122,123^
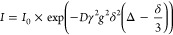
6where *I*_0_ is the signal intensity in the absence of a gradient, γ is the gyromagnetic ratio (γ_1H_ = 26.75 × 10^7^ rad T^–1^ s^–1^), δ is the gradient pulse length (2 ms), and Δ is the diffusion time (20–30 ms). The maximum gradient strength was adjusted from 200–1500 G cm^–1^, and the values of the other diffusion encoding parameters were selected to achieve ≥85% signal attenuation in 16 gradient steps.

The strong resonance from the acetyl methyl protons (2.06 or 1.18 ppm) was used for the diffusion measurements because all materials contain acetyl groups in approximately equal quantity. Chitosan diffusion coefficients were ∼10^–12^ m^2^ s^–1^ while coefficients for sulfated derivatives were ∼10^–11^ m^2^ s^–1^ (Table 2 and Figure S3). This again indicates that the sulfated derivatives are an order of magnitude lower DP than the starting chitosan, which is consistent with the reaction conditions and with molecular weight values found via viscometry and aqSEC.

The molecular weight estimates (Table 2) were calculated by converting the α value determined for chitosan to an *a* value through the relation: α = 3*a* – 1.^124^ A *k* value was extracted from eq 5 using the measured diffusion coefficient and *M*_w_ determined by aqSEC of OSC 0.77 or NSC 0.47 for *O*- or *N*-sulfated materials, respectively. The calculated *a* (0.75) and *k* (1.39 × 10^–7^ (*O*-sulfated) and 1.09 × 10^–7^ (*N*-sulfated) mL g^–1^) values were then used with the individual sample diffusion coefficients and eq 5 to determine the molecular weight of each material (see Table 2 for all molecular weight information).

### 2.4 CaCO_3_ Nucleation Experiments

#### 2.4.1 Preparation of Chitosan Surfaces for Nucleation Rate Measurements

The nucleation experiments required a stable substrate upon which the CaCO_3_ could form. Chitosan is insoluble at the experimental pH of 10 and remains a stable surface in solution without treatment (i.e., does not dissolve). However, the higher solubility of the sulfated chitosans (due to SO_3_^–^ groups) led to a surface that dissolved over time. These materials did not electrodeposit reliably, potentially due to the zwitterionic nature of the aqueous (pH 7) sulfated chitosan materials.

After considerable methods testing, sulfated chitosans were prevented from dissolving during the nucleation experiments by cross-linking (XL) each material with glutaraldehyde by adapting established methods.^125,126^ Our procedure began by preparing solutions of each chitosan material (2% w/v) and glutaraldehyde at a 4:1 ratio. Approximately 20 μL of the chitosan/glutaraldehyde cross-linked material was deposited onto gold-coated mica sheets (Platypus Technologies, ∼1 cm^2^) and dried overnight in a HEPA filtered oven (25 °C), resulting in a thin, insoluble film.

The degree of cross-linking cannot be accurately measured because glutaraldehyde can cross-link chitosan materials through the −OH and −NH (or −NH_2_) positions by various combinations, and the cross-links are dynamic in water. Therefore, the percentage of cross-linking in each chitosan material was estimated using relations developed for thermoset plastics.^127,128^ By this approach, the estimated value represents the theoretical maximum percent of cross-linking based on the concentrations of the chitosan material and glutaraldehyde and the number of active sites/reacting groups on the chitosan materials. We expect, however, the true value to be less than reflected by this estimate because of the dynamic nature of the XL bonds and because steric and viscosity effects likely prevent every site from being cross-linked. The reported values (Table 2) were calculated by the equation:
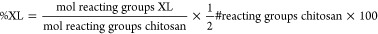
7

#### 2.4.2 Preparation of Solutions

Individual solutions of CaCl_2_ and Na_2_CO_3_ were prepared immediately prior to each nucleation experiment at concentrations that would achieve a desired supersaturation and a Ca^2+^ to CO_3_^2–^ activity ratio of approximately 1 upon mixing (Table S3). Calcium chloride dihydrate was used to prepare a 0.5 M CaCl_2_ stock solution, which was then diluted with degassed distilled/deionized water to prepare the solutions of CaCl_2._ For the Na_2_CO_3_ solutions, Na_2_CO_3_ (as powder) was weighed and dissolved in degassed DI water then adjusted to pH 10 using 1.0 M NaOH. Supersaturation (σ) with respect to the calcite polymorph of CaCO_3_ is defined as
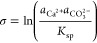
8where *a*_*i*_ is the activity of species *i* and *K*_sp_ the solubility product of calcite (10^–8.48^ at 25 °C).^129^ Geochemists Workbench was used to calculate the activity of each species from the solution compositions.^130^

#### 2.4.3 Measurement of Nucleation Rates

The rate of CaCO_3_ crystal nucleation onto chitosan-based surfaces was measured using an established flow through method^91,93,101^ which maintains a constant chemical driving force, σ, for the reaction over the duration of the experiment. Each nucleation experiment began by placing a substrate with chitosan material in an acrylic glass “reactor” (736 mm^3^) that was sealed with a glass coverslip to create a transparent imaging window. Solutions of CaCl_2_ and Na_2_CO_3_ (pH 10) were prepared and added to two polypropylene syringes (Sherwood Medical). These were mounted onto a high-precision syringe pump (PHD 2000, Harvard Apparatus) and connected to the chamber by Tygon tubing (1/16″ inner diameter, Cole Parmer) using a T-junction. For the first 10 min, solutions were dispensed at 20 mL h^–1^, following which the flow rate was reduced to 10 mL h^–1^ for the remainder of the experiment (up to 5 h). Preceding each experiment, DI water (pH 10) was flowed through the chamber over the polysaccharide surface at 20 mL h^–1^ for 25 min. Each experiment began using a new substrate and fresh material at a series of known supersaturations (4.61–5.74) and a constant temperature of 22 ± 1 °C.

**Figure 2:**
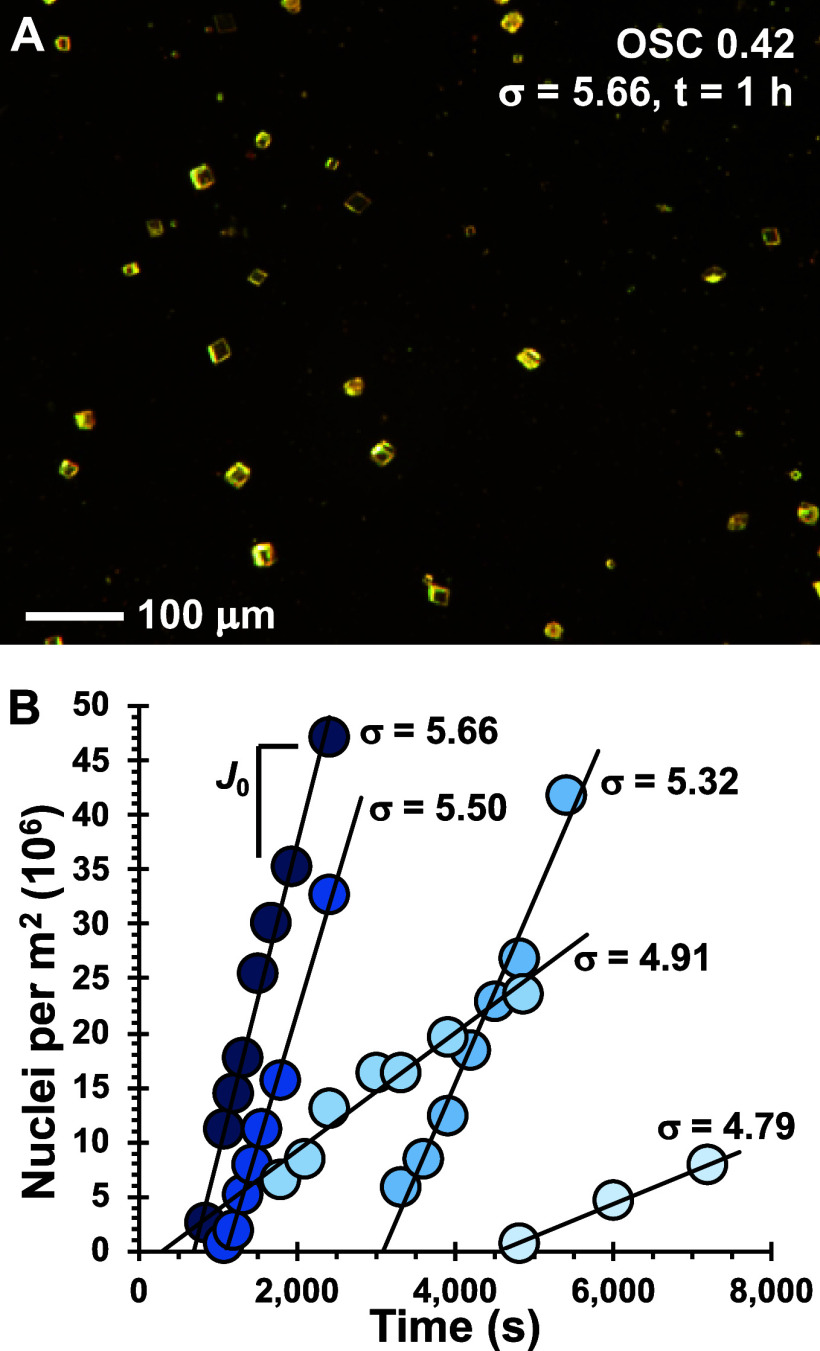
Representative kinetic data for chitosan material OSC DS(SO_3_^–^) = 0.42. (A) Optical image collected at 1 h of reaction time shows calcite crystallites (with postnucleation growth); (B) slope of the number of nuclei formed per area versus time is determined to obtain the nucleation rate, *J*_0_, from separate experiments conducted at each saturation state, σ (eq 8). The dependence of rate on 1/σ^2^ yields interfacial free energy, γ_net_ (e.g., eq 14 and Figure 3).

The crystallites that formed on each surface were imaged using the Z-stacking mode of a Zeiss AxioZoom.V16 microscope at a 50 or 100× magnification, making the viewing window 6.13 or 1.53 mm^2^, respectively (Figure 2A). By collecting a series of time-stamped images for data processing, the rate of nucleation was determined from the linear portion of the crystal density vs time data for each experimental condition of polysaccharide type and supersaturation. Data was collected as the number of nuclei per viewing window area per minute and was converted to SI units (number of nuclei per square meter per second) for data processing (Figure 2B).

Given that crystal nucleation occurs at a length scale below the resolution of the optical methodology, two assumptions were applied to interpret the measured rates using classical nucleation theory (see Section 3.1): (1) each crystal forms from a single calcite nucleus and (2) lateral interactions between crystallites and the local solution are minimal during the time interval where the increase in crystal density is linear and do not influence nuclei formation (e.g., Hu et al.,^101^ Giuffre et al.,^91^ Hamm et al.^93^).

All experiments were conducted at conditions where the input solutions (when combined) had calculated supersaturations that exceeded the solubility of amorphous calcium carbonate (ACC, σ = 4.61 with respect to calcite).^131^ However, there was no evidence of ACC formation in the experiments for any polysaccharide substrate. This observation is consistent with those in previous studies.^91,93^

#### 2.4.4 Characterization of Polysaccharide Surfaces and CaCO_3_ Crystallites

Representative polysaccharide surfaces were examined to determine the integrity of the substrates and the CaCO_3_ polymorphs that formed (Figure S4). Samples were prepared for imaging by adhering the mica substrate to aluminum specimen mounts (Ted Pella, Inc.) and coating with 6 nm Pt/Pd using a Leica EM ACE600 sputter coater. A JEOL IT-500HR analytical field emission gun-scanning electron microscope (FEG-SEM) was used to image the samples.

The CaCO_3_ polymorph(s) that formed were identified using a Rigaku MiniFlex II X-ray diffractometer (XRD) with a Cu tube. Spectra were collected at 30 K and 15 mA with a 0.02° step width and count time of 10 s. Calcite was the only polymorph detected during the data collection period (Figure S5).

### 2.5 Molecular Dynamics Simulations

Individual polymer chains were built with 19 glucosamine monomers using the PyMOL builder.^132^ With set probabilities and coordinates for different polymer compositions, a custom randomizer function in Python3^133^ was used to establish degree of acetylation (at C_2_N) and sulfation (at C_6_O or C_2_N) as well as monomer sequence. The resulting polymer compositions are given in Table 3. Note that no sulfates were placed on the C_3_O position for any material.

**Table 3:** Chitosan Compositions Investigated in MD Simulations

material^a^	DS(NSO_3_^–^)	DS(OSO_3_^–^)	DS(Ac)	DS(NH_2_)
chitosan	0	0	0.26	0.74
OSC 0.47	0	0.47	0.16	0.84
OSC 1.00	0	1.00	0.16	0.84
NSC 0.11	0.11	0	0.26	0.63
NSC 0.21	0.21	0	0.26	0.53
NSC 0.74	0.74	0	0.26	0
ONS 0.42	0.21	0.21	0.47	0.32
ONS 1.16	0.53	0.63	0.21	0.26

a The OSC and NSC notation denotes sulfation at the *O*- or *N*-position, respectively; ONS denotes sulfation is present at both *O*- and *N*-positions. The number that follows gives DS(SO_3_^–^) of the material.

Each experiment began by centering an individual polymer chain in a cubic box of side length 77 Å and added water to bulk density with the CHARMM-GUI Solution Builder.^134^ We then added ions Na^+^ (6.25 mM), HCO_3_^–^ (6.25 mM), Ca^2+^ (2.4 mM), and Cl^–^ (4.8 mM) using the Monte Carlo method,^135^ as implemented in CHARMM-GUI.

Each system was first run in GROMACS^136^ for 500 ns (2 fs time step) in the *NPT* ensemble (1 atm, 22 °C) using the nonpolarizable force field CHARMM36m.^137^ GROMACS trjconv^136,138^ was used to extract snapshots from the trajectory, saved every 100 ps, and to monitor the polymer end-to-end distance (using C_1_ of the first and last monomer, Figure S6) to establish the equilibrium conformation in the simulation cell.

The final GROMACS snapshot was used as input for further MD modeling with the AMOEBA polarizable force field,^139^ as implemented in the Tinker8 software package.^140^ These polarizable MD simulations were run in the *NPT* ensemble (1 atm, 22 °C) for 1 ns (1 fs time step), saving snapshots every 1 ps. Polymer end-to-end distances were calculated in each case to ensure that the polymer conformation was equilibrated (Figure S7). The AMOEBA trajectory was then used to calculate Radial Distribution Functions (RDFs) with VMD,^141^ enabling extensive analysis of atom proximity in the simulations.

## 3 Results and Discussion

### 3.1 Kinetics of CaCO_3_ Nucleation onto Derivatized Chitosans

The rate of calcite nucleation onto the chitosan controls and sulfated materials increases with increasing supersaturation as predicted by CNT (e.g., Figures 2B and S8). Nucleation occurs by the classical pathway of forming individual crystallites onto the polysaccharide materials without evidence of an amorphous intermediate. To estimate the energy barrier to nucleation for each calcite–polysaccharide system, we first determine the steady state rate of nucleation, *J*_0_, defined as
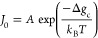
9where *A* is the kinetic prefactor, which includes the density of possible nucleation sites,^142^ attachment rates, and barriers to ion binding, such as the desolvation barrier;^143^*k*_B_ is the Boltzmann constant (J K^–1^), *T* is absolute temperature (K) and Δ*g*_c_ is the free energy barrier to forming a crystal nucleus of critical size (J mol^–1^). Δ*g*_c_ is given by
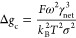
10where *F* is a nucleus shape factor (16/3π for a sphere)^144^ and ω is the molecular volume of the crystallizing phase (6.13 × 10^–23^ cm^3^ per molecule calcite).^145^ F and ω are assumed constant for a given polymorph. σ is supersaturation (eq 8), and γ_net_ (mJ m^–2^) is the interfacial free energy of forming calcite in the polysaccharide–solution system. Eq 10 shows that Δ*g*_c_ is dependent upon the inverse square of σ and the cube of the γ_net_.^146^ Substituting eq 10 into eq 9 yields
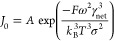
11

For constant *T*, we can simplify eq 11 by defining
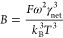
12

so that
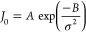
13where *B* contains γ_net_. Rewriting eq 13 into a linear form yields
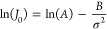
14

**Figure 3:**
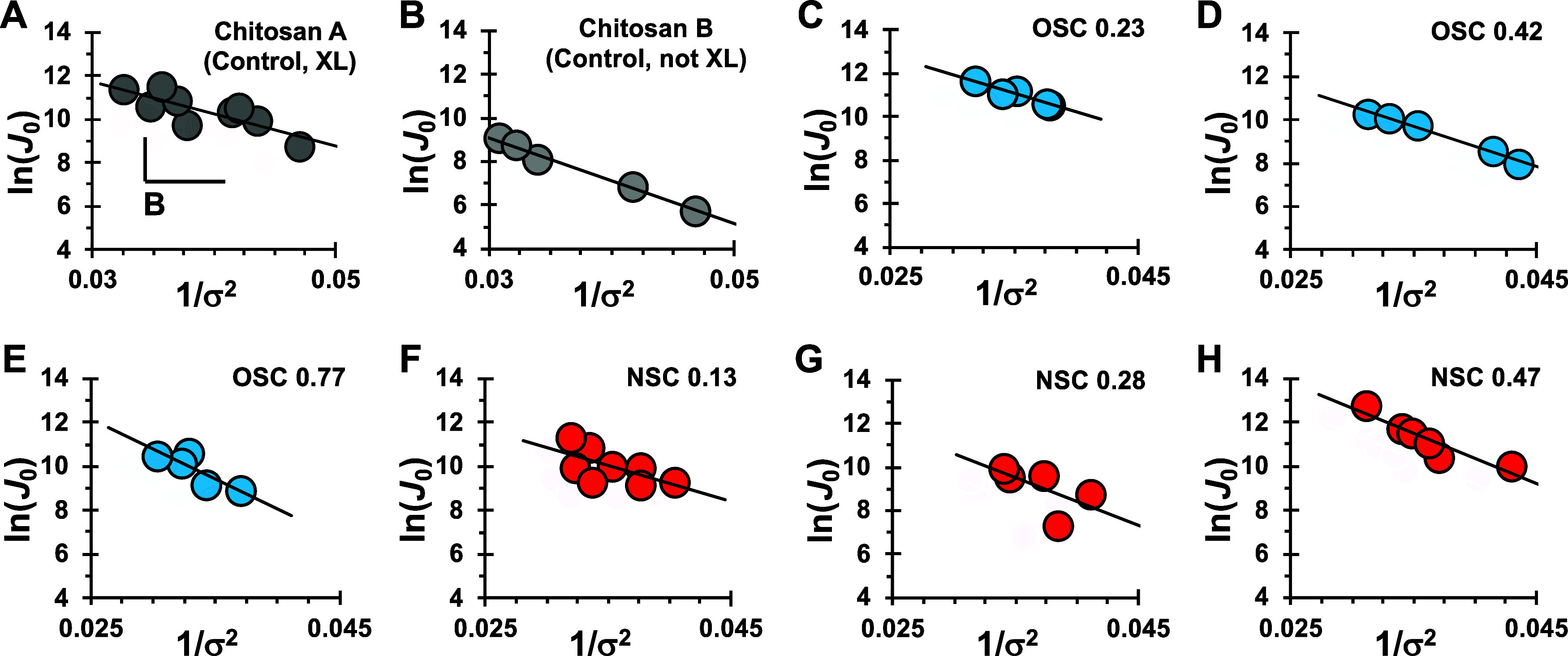
Experimental data show slope, *B*, is composition specific. Chitosan A and B are cross-linked (XL) and not-XL, respectively. The *B* values are determined for each experiment (±1 standard error) using eq 14: (A) *B*_chitosan A(Control, XL)_ = 148 ± 74; (B) *B*_chitosan B(Control, not XL)_ = 186 ± 11; (C) *B*_OSC 0.23_ = 171 ± 30; (D) *B*_OSC 0.42_ = 182 ± 10; (E) *B*_OSC 0.77_ = 273 ± 86; (F) *B*_NSC 0.13_ = 162 ± 81; (G) *B*_NSC 0.28_ = 217 ± 172; (H) *B*_NSC 0.47_ = 230 ± 47. All data were collected at pH 10, 22 °C.

Eq 14 predicts that the natural logarithm of the rate of crystal nucleation onto a given polysaccharide surface is linearly dependent on 1/σ^2^ at constant temperature (recall σ is constant for a given experiment). Plotting ln(*J*_0_) versus 1/σ^2^ for the rate data collected for each of the chitosan materials, Figure 3 shows a good fit of eq 14 for all polysaccharide materials.

### 3.2 Kinetic and Thermodynamic Parameters

**Figure 4:**
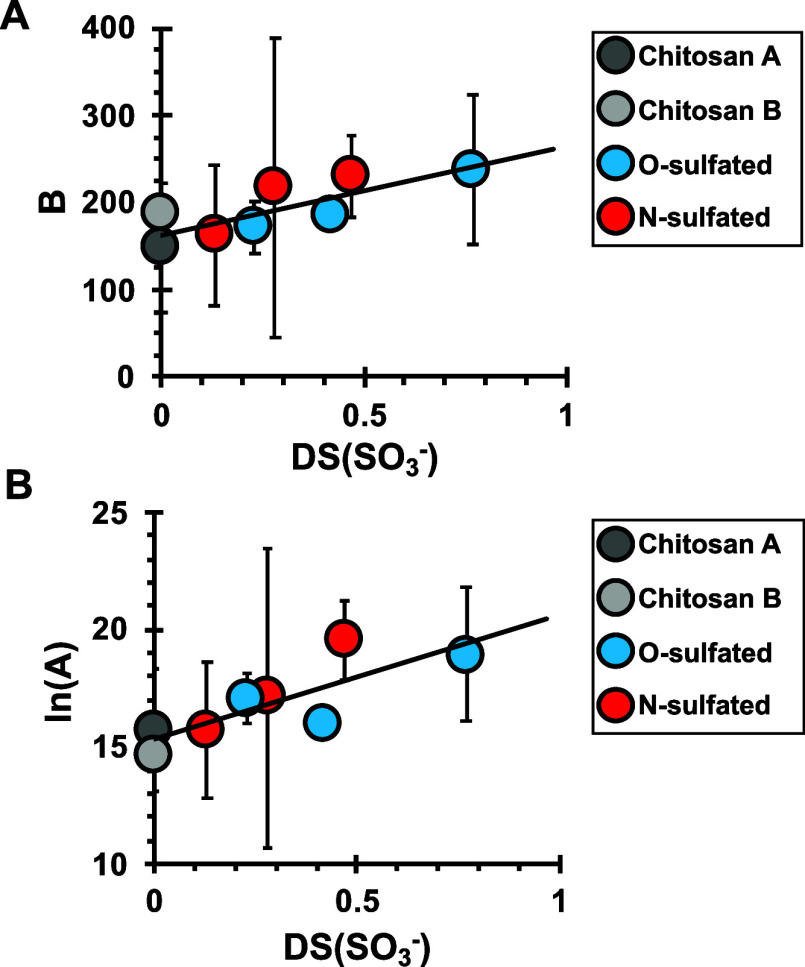
Analysis of kinetic data shows that (A) *B* (recall *B* ∝ γ_net_, eq 12) increases with sulfate density, DS(SO_3_^–^), but is otherwise approximately independent of composition. (B) Kinetic prefactor, *A*, also increases with DS(SO_3_^–^). The standard error of *B* and ln(*A*) are determined from the fit of eq 14 to the data in Figure 3.

Estimates of the thermodynamic (*B*) and kinetic (ln *A*) parameters determined from the experimental data (Table S4) illuminate three features of CaCO_3_ nucleation rate behavior onto sulfated chitosans. First, Chitosan A and Chitosan B (controls) present the lowest *B* values compared to their sulfated counterparts (Figure 4A). Their similarity demonstrates the energy barrier for nucleation onto chitosan is independent of the cross-linking protocol used in this study. Second, values of *B*, and hence γ_net_, are covariant with increasing DS(SO_3_^–^). Values of γ_net_ are independent of SO_3_^–^ position (*O*- or *N*-) on the chitosan molecule as seen by the single trend (Figure 4A). Nucleation of a new crystal is thus progressively less energetically favorable on increasingly charged materials as reported previously.^91,93^

A third feature is the 5–45× increase in the kinetic prefactor with increasing sulfation (Figure 4B) by a relationship that is independent of sulfate position. Terms within ln(*A*) are not readily evaluated, but the trend may reflect the larger number of sulfate sites available for interactions with Ca^2+^ at the chitosan–solution interface and/or higher rates of Ca^2+^ binding with SO_3_^–^ groups, as discussed below.^91,105^

#### 3.2.1 Components of the Free Energy Barrier to Nucleation

One expects the addition of sulfate groups onto chitosan to increase the number of sites for Ca^2+^–SO_3_^–^ complexation and thus increase the kinetic prefactor, ln *A*. However, the higher thermodynamic parameter contained in *B* that is associated with increasing DS(SO_3_^–^) is not readily understood. There are two possible explanations for this trend. First, we examine the γ_net_ term contained in *B* (eq 12) and recall that nucleating a new crystal onto a surface is controlled by contributions from three interfaces: calcite–solution (cal–soln), calcite–polysaccharide (cal–PS), and polysaccharide–solution (PS–soln) to give:
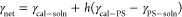
15where *h* is a nucleus shape factor. We approximate *h* to be constant given that γ_net_ varies by only 30% over the range of values determined from the experiments. For the conditions of this study, we also assume γ_cal–soln_ is approximately constant. Thus, increases in γ_net_ occur through an increasing γ_cal–PS_ and/or a decreasing γ_PS–soln_.

Giuffre et al. postulated that γ_PS–soln_ has primary control on how polysaccharide compositions modulate the kinetics of calcite nucleation due to strong water interactions with charged functional groups.^91^ By this explanation, reductions in γ_PS–soln_ are the primary driver of the higher γ_net_ associated with calcite formation onto sulfated and carboxylated polysaccharides. Our experimental findings are consistent with this interpretation—as derivatized chitosan materials become more hydrophilic with increasing sulfate density, the barrier for a crystal nucleus to displace water from sulfated groups becomes higher. Unfortunately, a simple charge density interpretation cannot provide further insight.

A second, related explanation for the trend in Figure 4A may be rooted in the nature of Ca^2+^ interactions with sulfate groups of the macromolecule and associated hydration properties. It is well-established that Ca^2+^ interacts with SO_4_^2–^ as solvent-separated and solvent-shared ion pairs in aqueous solution.^147−151^ Recent studies show solvent-separated interactions are also prevalent during the formation of amorphous calcium sulfate^151^ and hydrated CaSO_4_ salts such as gypsum.^151,152^ Macromolecule–cation interactions are also strongly influenced by hydration properties as evidenced by a calorimetric study that shows the binding of trivalent rare earth elements to carboxylated or phosphonated polymers is entropically driven by the release of hydration waters.^153,154^ MD simulations of Ca^2+^–SO_4_^–^ interactions also predict entropically driven effects caused by the associated release of water molecules.^148^

Density Functional Theory (DFT)^150^ and ab initio^149^ simulations predict the formation of solvent-separated Ca^2+^–SO_4_^2–^ ion pairs, with consequences for the primary and secondary hydration shells of calcium. Three observations of Ca^2+^–SO_4_^2–^ interactions by these investigators are relevant to this discussion: (1) an increase in the total hydration number about the ion pair (relative to the sum of the individual hydrated ions); (2) an increase in the rate of water exchange about associated cations; and (3) stronger interactions with increasing Ca^2+^ and SO_4_^2–^ ion concentrations.^149,150^ Reductions in the number of H-bonds within the solvation volume^150^ and reductions in the rate of water exchange in the region between calcium and sulfate^149^ are also noted. From these lines of evidence, we postulate that solvation interactions between Ca^2+^ and sulfate groups on the chitosan macromolecule may increase both γ_net_ and the frequency of ion binding.

### 3.3 MD Simulations of Sulfated Chitosan Environments

Using MD simulations, we examined the interaction of Ca^2+^ with a series of variably sulfated chitosan compositions and structures in an aqueous environment (see Section 2.5). In the initial model, free Ca^2+^ is coordinated by a maximum of 8 water molecules (7 with the AMOEBA force field). This is consistent with a previous study that compared multiple MD methods (LAMMPS, AMOEBA, and ab initio MD) to show that free Ca^2+^ is coordinated by approximatively 7 water molecules.^148^ As the MD simulation evolves and the ions interact with the different polymers, we find that Ca^2+^ in the CHARMM36 simulations (nonpolarizable) have between 6.5 and 8 coordinated water molecules. In the AMOEBA simulations (polarizable), Ca^2+^ coordinates 5.9–7.2 water molecules. The lower number of coordinated water molecules in our MD simulations suggests the Ca^2+^ ions interact with the polymer chain, replacing some of these waters with polymer-bound substituents (i.e., SO_3_^–^) in the system.

**Figure 5:**
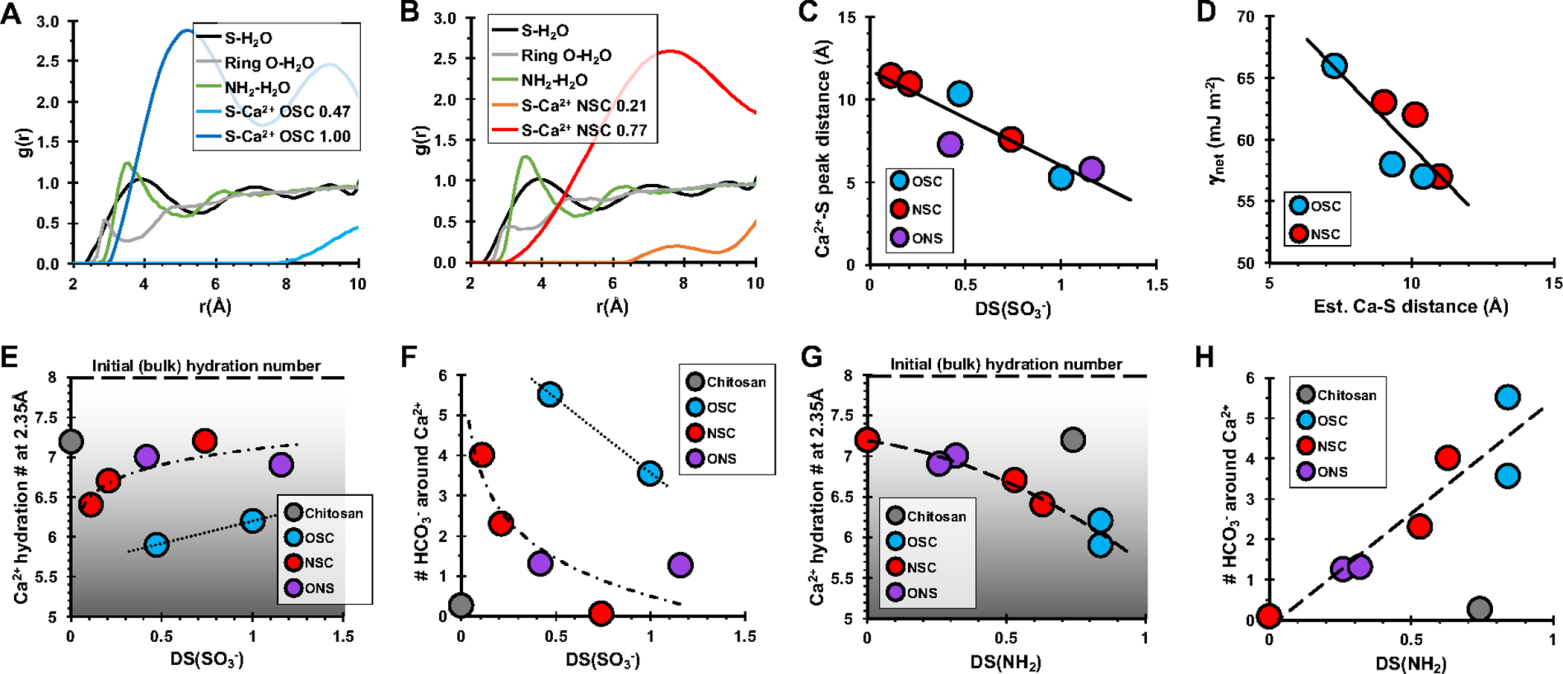
(A) RDF profiles for *O*-sulfated chitosans. Sulfate groups create a region of high-water structuring ≈3.9 Å of S–H_2_O separation (black) relative to uncharged substituents (gray). Charged amines also structure water (green). The model predicts Ca^2+^ ions are associated with sulfate in the lower-water density region ≈5 Å from the sulfate groups (compare blue and black lines), indicating solvent-separated Ca^2+^–sulfate interactions. A second region of Ca^2+^ is present ≈9 Å from sulfate groups. (B) RDF profiles for *N*-sulfated chitosans also show sulfate groups promote regions of high-water density. Ca^2+^ shows a greater separation distance of ≈7.5 Å from sulfate groups and a broader distribution (red). (C) Most probable distance between sulfated group and Ca^2+^ is inversely correlated with DS(SO_3_^–^), and independent of sulfate position. (D) Smaller Ca^2+^–S separation distance associated with the high DS(SO_3_^–^) is correlated with a high energy barrier to nucleation by a general relationship that is independent of sulfate position. (E) Number of waters about Ca^2+^ increase as DS(SO_3_^–^) increases (Ca^2+^–S separation declines), consistent with predictions of the solvation sphere about Ca^2+^–SO_4_^2–^ ion pairs.^148^ (F) Number of bicarbonate ions associated with Ca^2+^ decreases as DS(SO_3_^–^) increases but appears to be position-dependent. (G) Number of waters about Ca^2+^ decreases with increasing DS(NH_3_^+^) potentially due to a correlation with HCO_3_^–^. (H) Number of bicarbonate ions associated with Ca^2+^ increases as DS(NH_3_^+^) increases but this factor alone cannot explain all values.

The resulting RDF profiles give the probability, relative to a random distribution, of finding a reference atom at distance *r* from another reference atom (e.g., S–Ca^2+^, S–O of water). Figure 5A,B show RDFs for water about sulfate S, amine N, and the ring O atoms in chitosan and Ca^2+^ about sulfate S. By comparing the predictions for water and Ca^2+^ about the *O*-sulfated (Figure 5A) and *N*-sulfated (Figure 5B) molecules, the data provide three key insights into the molecular mechanisms at the origin of Ca^2+^ interactions in these systems.

First, sulfate and amine groups are associated with enhanced water structuring compared to water associated with other substituents of the polymer for all sulfated chitosan materials. In the simulations, amines are partially positively charged and present the highest charge density due to their small size relative to sulfate. This suggests water structuring about the polymer is directly proportional to the charge density of the substituents, which follows the order: amine *N* > sulfate S > ring O (the ring O is uncharged). For the experimental conditions used in this study (e.g., Section 2.4), the amine groups are not expected to be highly charged (≤+0.004 at pH 10),^99,113,155^ and we do not expect such pronounced water structuring around amine N atoms during nucleation rate measurements. Thus, we assume the water structuring about substituents in the experimental system has the order sulfate S > amine *N* > ring O.

The highest probability of finding a water molecule near an S atom is located at a S–H_2_O separation of ≈3.9 Å (Figure 5A,B, black lines). Given the relatively high charge density of each sulfate group, it is unsurprising that S–H_2_O probability profiles are independent of sulfate position (*O*- or *N*-linked). Our estimates of separation distance for these polysaccharide-bound sulfate groups are consistent with the 3.7–3.8 Å range determined for water structure about a free SO_4_^2–^ ion.^148,149^ Our results are also consistent with other computational studies reporting that sulfate groups promote long-range water structuring^156,157^ and increase the hydrogen bond density between polysaccharides and water.^158^ Sulfate groups on chitosan may also create hydrogen bonds between sulfate and nitrogen functional groups via a water molecule as reported for dermatan sulfates.^158^

Second, Ca^2+^ ions are solvent-separated from sulfate groups at the *O*- or *N*-positions (Figure 5A,B). The absence of contact ion pairs concurs with studies showing that Ca^2+^ and SO_3_^–^ interact as solvent-separated ion pairs.^148−150,159^ For example, RDF profiles for the *O*-sulfated chitosan (OSC 1.00) show Ca^2+^ is solvent-separated, at ≈5 Å from sulfate groups, which overlaps with the region of minimum water density (compare dark blue and black lines in Figure 5A). A second population of Ca^2+^ is predicted at ≈9 Å (Figure 5A). The RDF for *N*-sulfated chitosan (NSC 0.77) shows a broader distribution with most Ca^2+^ found at ≈7.5 Å separation from S (Figure 5B). The different Ca^2+^–S profiles in Figure 5A,B may be due to the greater conformational freedom of sulfates at the C_6_O-position of the *O*-sulfated chitosan (e.g., Figure 1A_ii_) compared to the steric hindrance associated with sulfates at the C_2_N-position of the *N*-sulfated chitosan (e.g., Figure 1A_iii_). S–Ca^2+^ RDF profiles in a chitosan environment that presents sulfate groups at both the *O*- and *N*-positions (e.g., Table 3, ONS) are not well-resolved (Figure S9), an effect likely reflecting interaction of Ca^2+^ with sulfate groups at both positions.

Third, the Ca^2+^–S separation distance is inversely correlated with increasing DS(SO_3_^–^) for all materials (Figure 5C). To obtain this relation, we use the RDF profiles to estimate the average distance between each S atom and the most probable population of Ca^2+^ ions near the sulfate groups (i.e., location of maximum). For ONS materials, the distance corresponding to the first maximum was used (Figure S9). The trend is independent of sulfate position which suggests local substituents about sulfate do not significantly affect the separation distance for these Ca^2+^–S interactions. Interestingly, if we consider the closest calcium probability to S atoms (i.e., when *g*(*r*) is first >0), the separation distance declines to a near-constant value of ≈3.5 Å (Figure S10), further indicating solvent-separated binding.^160^

### 3.4 A Closer Look at Solvation as a Lever for Modulating Nucleation

The experimental and computational evidence suggest the influence of sulfation of chitosan on nucleation can be understood as a macroscopic consequence of competing interfacial energies through the changing hydrophilicity of the macromolecule. Figure 5D compares experimental estimates of γ_net_ with calculated Ca–S separation distances for corresponding DS(SO_3_^–^). At first glance, the inverse correlation of a larger γ_net_ to forming a new crystal with smaller Ca–S separation seems contradictory. As separation distance declines, one expects a decrease in γ_cal–PS_ that pushes γ_net_ in the opposite direction of the trend determined from experiment. We postulate this is offset, however, by the large increase in hydrophilicity with sulfation of the chitosan macromolecule. This stabilizes the polysaccharide-water interface and lowers γ_PS–soln_ to increase γ_net_ (eq 15). Stated in physical terms, it is more difficult to displace water from the increasingly hydrophilic macromolecule.

Further examination of the model predictions suggests that an explanation based solely on charge density is incomplete. Figure 5E shows the number of waters about Ca^2+^ near sulfate increases as the local environment becomes progressively more hydrophilic (Figure 5E). However, the trend for chitosan materials that have SO_3_^–^ at the *N*-position is offset from those with SO_3_^–^ at the *O*-position. Similarly, the number of HCO_3_^–^ ions associated with Ca^2+^ decreases with DS(SO_3_^–^), again with an offset for the *O*-sulfated versus the other materials (Figure 5F). These results suggest a progressive competition between SO_3_^–^ and HCO_3_^–^ for Ca^2+^ that potentially increases γ_net_ through decreasing calcium–carbonate binding en route to forming critical nuclei. However, the experimental estimates of γ_net_ or the kinetic prefactor, ln *A* (e.g., Figure 4A,B) do not show regiospecific trends. It is possible the differences are smaller than the resolution of the kinetic measurements or that the model results are incorrect.

An alternative explanation is that the material-specific offsets in Figure 5E,F indicate charged amines also have a role in Ca^2+^–H_2_O–HCO_3_^–^ interactions. Recall the sulfation of nitrogen groups (DS(NSO_3_^–^)) corresponds to a 1:1 reduction in DS(NH_2_) (e.g., Table 3, NSC, ONS), whereas DS(NH_2_) remains constant when sulfate is added to the chitosan *O*-positions. Figure 5G indicates the number of hydration waters about Ca^2+^ declines with increasing DS(NH_2_) to give a single trend that is independent of sulfate position. The trend is covariant with an increasing number of HCO_3_^–^ molecules around Ca^2+^ for increasing DS(NH_2_) (Figure 5H). The model relations thus suggest that offsets in Figure 5C,D trends are reconciled by an interplay with amines, while inconsistencies between materials of the same DS(NH_2_) (e.g., OSC 0.47, OSC 1.00) can be rationalized by differences in DS(SO_3_^–^). This would indicate both the sulfate and amine functional groups significantly influence how H_2_O and HCO_3_^–^ interact with Ca^2+^ at the polysaccharide–water interface in the simulation environment.

The model results for chitosan in Figure 5G,H are consistent with this interpretation. In the absence of sulfate groups, chitosan has weak Ca^2+^–polysaccharide interactions, suggesting that chitosan (with or without charged amines) will result in the slowest rate of CaCO_3_ nucleation compared to the sulfated materials due to the low reaction frequency. The low γ_net_ experimentally determined for chitosan can be understood by recognizing that chitosan has little charge at pH 10, which leads to low hydrophilicity. Taken together, these results raise exciting questions regarding how cooperative interactions of positive (amines) and negative (sulfate) charged groups can modulate the crystallization of inorganic materials onto functionalized macromolecules.

### 3.5 Dependence of γ_net_ on Charge per Monosaccharide

**Figure 6:**
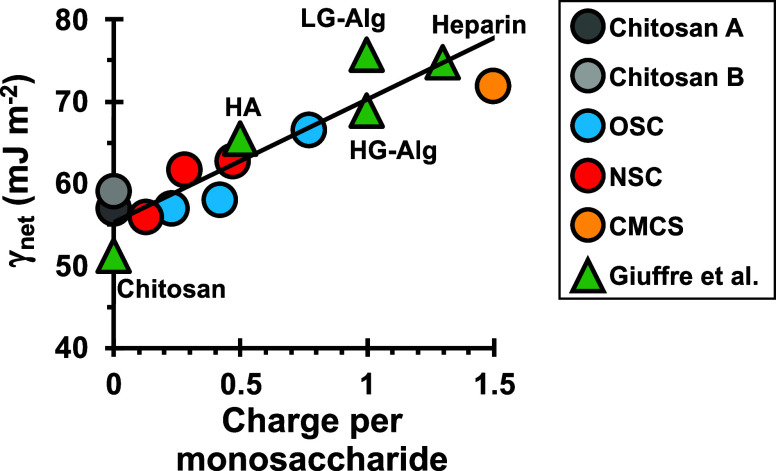
Energy barrier to calcite nucleation correlates with net charge per monosaccharide by a general relationship that includes diverse polysaccharide compositions. The dependence includes values reported by Guiffre et al.,^91^ for heparin, hyaluronic acid (HA), and two alginates with high (HG-Alg) or low (LG-Alg) guluronic acid content. Tests of carboxymethyl chitosan (CMCS, Figure 7) also agree with the trend. Values of γ_net_ (mJ m^–2^) shown here: γ_net Chitosan A(Control, XL)_ = 57; γ_net Chitosan B(Control, not XL)_ = 59; γ_net NSC 0.13_ = 54; γ_net NSC 0.28_ = 62; γ_net NSC 0.47_ = 63; γ_net OSC 0.23_ = 57; γ_net OSC 0.42_ = 58; γ_net OSC 0.77_ = 66; and γ_net CMCS_ = 72. And: γ_net Chitosan(Giuffre)_ = 51; γ_net HA(Giuffre)_ = 65; γ_net HG-Alg(Giuffre)_ = 69; γ_net LG-Alg(Giuffre)_ = 76, γ_net Heparin(Giuffre)_ = 75. Values were obtained using eq 12 and *F* = 16/3π, ω = 6.13 × 10^–23^ cm^3^, and *T* = 22 and 25 °C for data reported in this study and Giuffre et al.,^91^ respectively.

By compiling γ_net_ values reported by previous investigations and those determined in this study, Figure 6 shows all sulfated chitosan materials (this study) obey a single trend that also includes uncharged, sulfated, and carboxylated materials. The relationship raises the question of whether solvation about sulfate groups can also explain the kinetics of calcite nucleation onto carboxylated polysaccharides.

**Figure 7:**
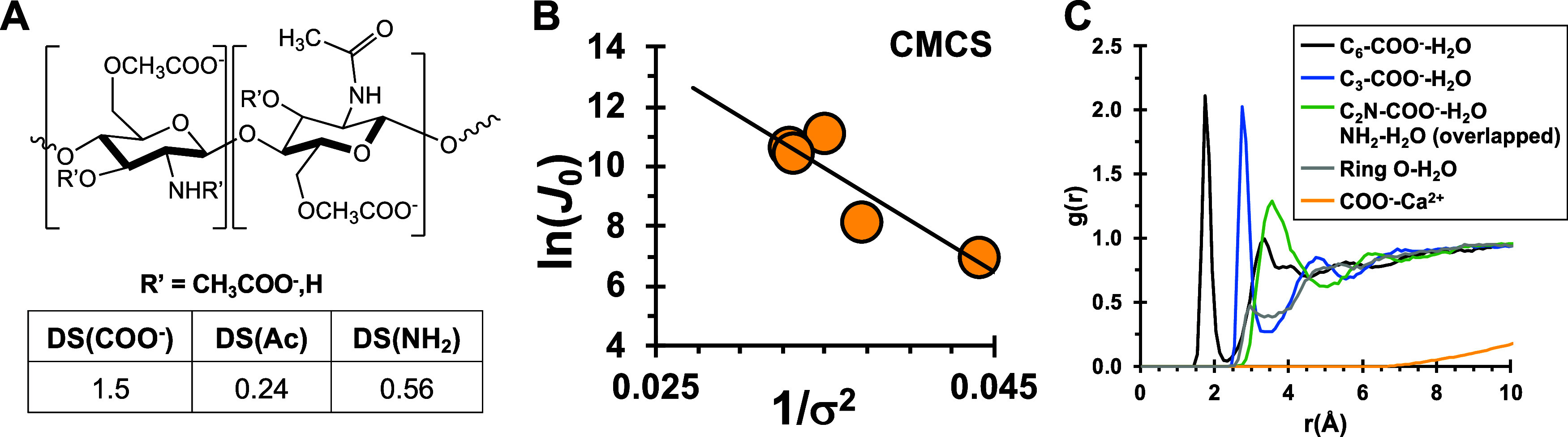
(A) Carboxymethyl chitosan (CMCS, prepared and characterized by Zhou et al.).^108^ (B) Calcite nucleation rate measurements yield *B*_CMCS_ = 346 ± 99. (C) MD simulations show carboxyl groups also create regions of high-water structuring with a magnitude that is greater than sulfate but dependent on carboxyl position.

To test this idea, we performed calcite nucleation experiments and MD simulations using carboxymethyl chitosan (CMCS). This polysaccharide is thus analogous to the sulfated chitosan derivatives, presenting COO^–^ groups at the C_6_O-, C_3_O-, and C_2_N-positions (Figure 7A). Rate measurements for CMCS obtained γ_net,CMCS_ = 72 mJ m^–2^ (Figure 7B and Table S4). This value is lower than but in general agreement with the trend in Figure 6. As predicted by our model, RDF profiles for CMCS indeed indicate more water structuring about the three carboxyl positions relative to uncharged substituents of the polymer (Figure 7C). The simulation also indicates carboxyl–water interactions depend strongly on position whereby C_6_O–CH_2_COO^–^ and C_3_O–CH_2_COO^–^ have the highest water structuring compared to C_2_N–CH_2_COO^–^ (Figure 7C). The H_2_O–COO^–^ distances are also position-specific with high probabilities at ≈1.7, 2.7, and 3.5 Å, respectively. The model predicts Ca^2+^–COO^–^ interactions are weak and remain solvent separated.

The single trend in γ_net_ vs charge per monosaccharide for sulfated and carboxylated materials (Figure 6) supports the physical model that nucleation rate is an interplay between the interactions of Ca^2+^ with charged groups and the increasing hydrophilicity associated with greater charge density. For polysaccharides where Ca^2+^ interactions are solvent-separated, such as for sulfated and carboxylated macromolecules, we concur with the explanation that γ_net_ is a physical result of charged groups regulating nucleation through reductions in γ_PS–soln_ that overpower the reductions in γ_cal–PS_.^91^ The relations also suggest these groups have similar effects on rates of calcification.

Our findings raise the question of how macromolecules would be expected to influence values of γ_net_ and A if calcite nucleation occurred via a nonclassical process involving amorphous calcium carbonate (ACC). Many CaCO_3_ biominerals are now recognized to form by nonclassical processes, often beginning with ACC. Assuming that (1) the ACC is hydrated, unstructured, and equilibrated with the associated solution Ca^2+^ and bicarbonate ion concentrations, and (2) Ca^2+^-sulfated macromolecule interactions are similar to those predicted in this study, then we would expect γ_net_ and *A* values to scale with charge density similar to what is reported herein. Unfortunately, to our knowledge, this cannot be readily tested. It is experimentally implausible to vary saturation state with respect to ACC on a time scale that allows estimates of γ_net_ (or *A*) for the ACC to crystalline nucleation process. The influence of macromolecule composition on crystal nucleation via nonclassical pathways is a topic that warrants exploration.

## 4 Conclusions

This experimental study of CaCO_3_ nucleation onto a series of sulfated chitosan materials shows that the free energy barrier to calcite nucleation (Δ*g*_*c*_) increases with the density of sulfate groups of the macromolecule through controls on the interfacial free energy of the calcite–polysaccharide–solution system (γ_net_). Materials with larger DS(SO_3_^–^) correlate with higher γ_net_, likely through reductions in lower γ_PS–soln_. Thus, γ_net_ is strongly dependent upon the density of SO_3_^–^ groups but independent of sulfate position within the 2-amino-2-deoxyglucose monosaccharide of the chitosan macromolecule. Parallel MD simulations of Ca^2+^–water interactions with three types of sulfated compositions support the physical model for a relationship between the degree of sulfation and increasing hydrophilicity of the macromolecule. Greater DS(SO_3_^–^) results in progressively closer, albeit solvent-separated, Ca^2+^–SO_3_^–^ interactions with associated increases in hydration of the calcium–sulfate ion pair.

These findings raise two points regarding CaCO_3_ nucleation onto chitosan and the influence of sulfate functional groups. First, although sulfation increases the free energy barrier to forming calcite, sulfated chitosan compositions can nonetheless be good nucleators through increases in the kinetic prefactor. At low supersaturation, the increased γ_net_ due to the greater hydrophilicity of the macromolecule wins out and nucleation is inhibited. However, at sufficiently high supersaturation and sulfate density, the inhibitory effect of the higher γ_net_ is overwhelmed by increases in the pre-exponential term of the rate expression to give faster nucleation. The model results suggest this is caused by disruptions in the local water structure about Ca^2+^. Second, our study underscores the importance of solvation about ions and the polysaccharide–solution interface in the nucleation of sparingly soluble crystalline materials. This appears especially significant for macromolecules with anionic groups that have solvent separated interactions with the cation of the nucleating material. The general relationship between γ_net_ and net charge per monosaccharide for sulfated and carboxylated polysaccharides (Figure 6) leads us to conclude carboxyl and sulfate groups have similar roles in CaCO_3_ nucleation. We predict that a variety of Ca-bearing and possibly other alkaline earth salts may also obey this trend.

More broadly, the relationships reported herein for diverse polysaccharides raise the question of whether polymer-bound functional groups, as individuals or cooperatively, are the overarching players in biological crystallization. The long-standing focus of the biomineralization community on carboxylated proteins in biological mineralization has provided tremendous insight. However, given that sulfate groups are widely associated with polysaccharides at sites of CaCO_3_ biomineralization in animals and algae,^26^ we suggest this perspective may be incomplete. Perhaps the configurations and motifs of functional groups, in combination with their associated solvation environments, are the primary drivers of crystallization, irrespective of macromolecular class.

The findings also suggest that chitosan composition can be tailored to present desired structure–property relationships for modulating crystallization of CaCO_3_, and possibly other sparingly soluble salts, to create synthetic biocomposites for specialized applications. Using the type, density, and position of functional groups to adjust the thermodynamic and kinetic levers that control the onset of crystallization, it may be possible to produce complex assemblies onto and within 2D and 3D printed hydrogels composed of differently functionalized biopolymers. Recent innovations that selectively deposit soft materials at high resolution^161^ are making such multifaceted applications possible.

## Supporting Information Available

Summary of selected studies of CaCO_3_ nucleation onto or in the presence of sulfur-containing macromolecules; synthesis conditions; nucleation experiment solution compositions; *B*, ln(*A*), and γ_net_ values for each material; determination of chitosan intrinsic viscosity; aqueous SEC chromatograms for OSC 0.77 and NSC 0.47; determination of chitosan diffusion coefficient; CaCO_3_ SEM image; CaCO_3_ XRD; polymer end-to-end distances Charmm; polymer end-to-end distances amoeba; nuclei vs time data from nucleation experiments; RDF profiles for *N*- and *O*-sulfated materials (ONS 0.42, 1.16); correlation between nearest S–Ca^2+^ distance and DS(SO_3_^–^); estimated closest S–Ca^2+^ distances for experimental materials and correlation to determined γ_net_ values (PDF)
