# Supplementary material for: Kinetics of Calcite Nucleation onto Sulfated Chitosan Derivatives and Implications for Water–Polysaccharide Interactions during Crystallization of Sparingly Soluble Salts
Source: Cryst Growth Des. 2024 Jul 11;24(15):6338–53. doi: 10.1021/acs.cgd.4c00602 (PMC11311137; doi:10.1021/acs.cgd.4c00602)
Supplement: Supplementary file 1 — cg4c00602_si_001.pdf [file cg4c00602_si_001.pdf]

Supporting Information

Kinetics of calcite nucleation onto sulfated chitosan derivatives and implications for water-polysaccharide interactions during crystallization of sparingly soluble salts

Brenna M. Knight, Ronnie Mondal, Nizhou Han, Nicholas F. Pietra, Brady A Hall, Kevin J. Edgar, Valerie Vaissier Welborn, Louis A. Madsen, James J. De Yoreo, Patricia M. Dove

Table S1: Summary of Selected Studies of $CaCO_3$ Nucleation onto or in the Presence of Sulfur-Containing Macromolecules **(Pg. 2)**

Table S2: Synthesis Conditions **(Pg. 8)**

Table S3: Nucleation Experiment Solution Compositions **(Pg. 9)**

Figure S1: Determination of Chitosan Intrinsic Viscosity **(Pg. 10)**

Figure S2: Aqueous SEC chromatograms for OSC 0.77 and NSC 0.47 **(Pg. 11)**

Figure S3: Determination of Chitosan Diffusion Coefficient **(Pg. 12)**

Figure S4: $CaCO_3$ SEM Image **(Pg. 13)**

Figure S5: $CaCO_3$ XRD **(Pg. 14)**

Figure S6: Polymer end-to-end distances Charmm **(Pg. 15)**

Figure S7: Polymer end-to-end distances Amoeba **(Pg. 16)**

Figure S8: Nuclei vs Time data from nucleation experiments **(Pg. 17)**

Table S4: B, ln(A), and γ_{net} Values for Each Material **(Pg. 18)**

Figure S9: RDF profiles for *N*- and *O*- sulfated materials (ONS 0.42, 1.16) **(Pg. 19)**

Figure S10: Correlation between nearest S-Ca^{2+} distance and DS(SO_3^-); estimated closest S-Ca^{2+} distances for experimental materials and correlation to determined γ_{net} values. **(Pg. 20)**

References (Pg. 21)

Table S1: Summary of Selected Studies of $CaCO_3$ Nucleation onto or in the Presence of Sulfur-Containing Macromolecules

Material	$CaCO_3$ Polymorph and Morphology	Method and Notes	Ref.
**Polysaccharides**			
Dermatan sulfate	Calcite, small columnar	Observational, gas diffusion method, crystals were generally smaller and more numerous than with no treatment	1
Dermatan sulfate (de-sulfated)	Calcite, rounded rhombohedral		
Heparin	Rosette aggregates, polymorph unspecified		
Heparin (low sulfation)	Calcite, twinned		
Heparin	Calcite, rough rhombs	Observational, ammonium carbonate method, crystals became porous as time increased from 1 day to 7 days	2
Heparin (low molecular weight)	30:70 calcite:vaterite, Irregular rhombs and spheres	Observational, direct mixing, crystal size decreases and deformation increases with concentration of heparin	3
Heparin	$CaCO_3$, polymorph unspecified	Observational, direct mixing, pH 9.75, authors suggest neutral polymers stabilize pre-nucleation clusters and charged polymers also stabilize but can't determine between pre and post nucleation	4
Dextran sulfate	$CaCO_3$, polymorph unspecified		
Keratan sulfate (avian derived)	ACC[1], vaterite (later transformed to calcite)	Quantitative; potentiometric titration, stabilization of prenucleation clusters observed, greater inhibition by keratan sulfate than dermatan sulfate	5
Dermatan sulfate (avian derived)	ACC[1]		
Chondroitin-4-sulfate	Vaterite, 77% Calcite, 23% Spheres and rhombs	Observational, direct mixing	6
Chondroitin-4-sulfate	Vaterite	Quantitative, constant composition method, $\gamma = 46$ mJ m^{-2}	7
Chondroitin-6-sulfate	Vaterite	$\gamma = 62$ mJ m^{-2}	
Dermatan sulfate	Vaterite	$\gamma = 49$ mJ m^{-2}	
κ-carragenan	Calcite, intermediate between stacked and rosette crystals	Observational, direct mixing, compared to other polysaccharides, morphology based on if material formed a gel	8
κ-carragenan	Calcite, porous rhombs	Observational, direct mixing with a double jet reactor (constant flow in but not out), all carrageenan materials increased induction and crystallization time	9
ι-carragenan	Calcite, porous rhombs, faceted		
λ-carragenan	Calcite, porous rhombs, faceted		
Dextran	Aragonite needles	Observational, direct mixing method, pH 6	10
Dextran sulfate	Calcite and vaterite spheres	Phase separation of polymer observed	

Polysaccharide from *E. Huxleyi* coccolith (sulfated and carboxylated)	Calcite, induction time inhibited compared to solution alone	Observational, direct mixing/ titration, hypothesize sulfate groups are in the interior of the molecule rather than the surface or bound to cations, conclude carboxyl groups play a larger role in inhibition	11
Polysaccharide from *E. Huxleyi* coccolith (95% desulfated)	Calcite, induction time inhibited, no change from control		
Polysaccharide from *E. Huxleyi* coccolith (80% decarboxylated)	Calcite, no inhibition		
Chitosan/ ι-carrageenan composite	$CaCO_3$, polymorph unspecified	Observational, direct mixing, composite dipped in biopolymer solution then immersed sea water (SSW)	12,13
chitosan/ ι-carrageenan with PAA[2]	$CaCO_3$, polymorph unspecified	Rates increased relative to control	
chitosan/ ι-carrageenan with citric acid	$CaCO_3$, polymorph unspecified	Rates increased relative to PAA[2] (and control)	
chitosan/ ι-carrageenan with glutamic acid	$CaCO_3$, polymorph unspecified	Suggest acidity increases crystallization rate, glutamic>citric>PAA	
Chitosan and chondroidin-4-sulfate	Vaterite, 77% Calcite, 23% Rough spheres	Observational, direct mixing, suggest chain rigidity influences morphology	6
**Proteins + protein/ polysaccharide mixtures**			
Eggshell proteins of *G. domesticus* (chicken)	Calcite, columnar, fused	Observational*, in vivo*, untreated	14
Sulfation of eggshell proteins inhibited	Calcite, spherical + short, poorly fused	Observational*, in vivo*, sodium chlorate treatment to inhibit sulfation of eggshell proteins	
Demineralized eggshell membranes of *G. domesticus* (chicken) containing sulfur residues	Calcite rhombs associated with sulfur containing areas	Observational, ammonium carbonate method	15
Demineralized eggshell membranes of *G. domesticus* (chicken) with sulfur removed	Calcite, random distribution		
Demineralized eggshell membranes of *G. domesticus* (chicken) containing sulfur residues and crude organic matrix extract from *G. domesticus* (chicken)	Calcite, modified and smaller than with eggshell membrane alone	Observational, ammonium carbonate method, crystals localized around sulfur "patches"	
Demineralized eggshell membranes of *G. domesticus* (chicken) containing sulfur residues and isolated dermatan sulfate proteoglycan from *G. domesticus* (chicken)	Calcite, modified, smaller and rounder than with crude organic matrix		
Acidic proteins from *M. californianus* (bivalve)	Calcite, 14.6% of total crystallites	Observational, ammonium carbonate diffusion	16

	oriented onto substrate		
Proteins from *U. pictorum* (bivalve)	Calcite aggregates, delayed nucleation	Observational, gas diffusion method, protein highly sulfated, complete inhibition at higher concentrations of protein	17
Deglycosylated proteins from *U. pictorum* (bivalve)	Calcite, deformed, no inhibition		
Nautilin-63, glycoprotein of *N. macromphalus* (mollusk)	Calcite, deformed	Observational, gas diffusion method, Nautilin-63 is carboxylated and sulfated, deformation increased with concentration of protein	18
Chitin and soluble OM from *A. Psittacus* (barnacle)	Vaterite that transformed to rounded calcite, then rhombohedral	Observational, gas diffusion method, deproteinization of chitin led to some deacetylation	19
Isolated proteoglycans from *A. Psittacus* (barnacle)	Calcite, modified, piles with c-axis alignment		
Insoluble OM (containing chitin) from *A. Psittacus* (barnacle)	Calcite with c-axis orientation	Observational, direct mixing, insoluble organic matrix contains proteoglycans	20
Soluble OM from *A. Psittacus* (barnacle)	Calcite, altered and aggregates	Soluble organic matrix contains non-specific sulfates	
rOC90[3]	Calcite, elongated rhombs	Observational, ammonium carbonate method, modeling suggests tertiary structure clumps anionic sections of molecule	21
**Synthetic**			
PSS[4]	Calcite, 3-7.5% of total crystallites oriented onto substrate	Observational, ammonium carbonate method, % calcite formed correlates with degree of sulfation (to a plateau)	16
PSS[4] and poly(aspartic acid)	Calcite, 2-25% of total crystallites oriented onto substrate	Observational, ammonium carbonate method, % affected by exposure time	
PSS[4]	ACC[1]	Observational, gas diffusion method, nucleation inhibited in bulk solution	22
PSS[4]	ACC[1] that transforms to round, porous calcite	Observational, gas diffusion method, crystals get rounder as PSS[3] or Ca^{2+} inc.	23,24
PSS[4] (low conc.)	Calcite, rhomb and vaterite, spherical, heterogeneous particle size	Observational, direct mixing, suggest sulfonate groups block CO_3^{2-} binding sites, induction time much higher than control at low saturation	25,26
PSS[4] (high conc.)	Vaterite, spherical and homogenous particle size		
PSS[4]	Vaterite, spheres	Observational, direct mixing, spheres composed of nanocrystals	27
PSS[4]	Calcite, spherical	Observational, ammonium carbonate method, later added ethanol to mixture which inc. vaterite content	28

PSS[4]	Vaterite, smooth spheres	Observational, direct mixing, suggest PSS[4] complexes Ca^{2+} to block sites and repel CO_3^{2-}	29
PSS[4]	Calcite/ vaterite mixture, spheres	Observational, direct mixing, size of spheres increased with $CaCO_3$ conc. until threshold past which rhombs formed/ no PSS[4] effect	30
PSS[4]	Vaterite, uniform spheres	Observational, direct mixing, suggest PSS[4] reduces driving force through interfacial energy and phase-stabilizes vaterite. High Ca^{2+}:CO_3^{2-} ratio promoted vaterite more than low	31
PSS[4]	Vaterite, 82%<br>Calcite, 18%	Observational, rapid mixing, cauliflower shape until critical concentration, then spherical morphology	32
PVS[5]	Vaterite, 86%<br>Calcite, 14%		
PSS[4] and polystyrene-polyethylene oxide copolymer	Vaterite, rough spheres	Observational, direct mixing, copolymer forms micelles that nuclei form within leading to competition between complexation and adsorption	29
PSS[4] + CTAB[6]<br>(low conc.)	Calcite, rough spheres	Observational, direct mixing, hypothesize CTAB[6] changes PSS[4] morphology, reducing effect	
PSS[4] + CTAB[6]<br>(high conc.)	Calcite, aggregated and faceted rhombs	Observational, direct mixing, Size of spheres increased with $CaCO_3$ conc. until threshold past which rhombs formed/ no PSS[4] effect	
PSS[4] and PolyDADMAC[7]	Vaterite majority with some calcite and aragonite	Observational, direct mixing, cauliflower shape until critical concentration, then spherical morphology, % aragonite increased with concentration polyDADMAC[7]	32
PVS[5] and PolyDADMAC[7]	Vaterite majority with some calcite and aragonite		
PVS[5]	Vaterite, spheres	Observational, direct mixing, suggest PVS[5] slows rate by sequestering Ca^{2+} and stabilizes vaterite, increased aggregation at high PVS[5] conc.	33
PSS[4]	Vaterite, uniform spheres	Observational, direct mixing, crystals became angular with time in the presence of PAMPS[8]	34
PAMPS[8]	Vaterite, aggregated 'near' spheres		
PAMPS[8]-co-PAA[2]	Vaterite, 93%<br>Calcite, 7%<br>spheres	Observational, direct mixing	6
PAMPS[8]-co-PAA[2]	Vaterite, 92.5%<br>Calcite, 7.5%	Observational, direct mixing, smaller, more numerous crystals as concentration of PAMPS[8]-co-PAA[2] increases	35
Sulfated PMS[9] in solution	Calcite, rounded rhombs	Observational, gas diffusion method, c-axis alignment and increased deformation as concentration S-PMS[9]	36
Sulfated PMS[9] film	Calcite, rounded rhombs, selective nucleation based on chain positions	Observational, gas diffusion method, no c-axis alignment	

PAMPS[8]	Calcite and aragonite, oriented triangular and tubular	Observational, gas diffusion method	37
PAMPS[8]-co-acrylamide hydrogel	Calcite, aggregates and cuboctahedral	Observational, direct mixing, poly(acrylamide) alone gives pseudo-octahedral aggregates	38
Polymer with gradient amine content	Calcite, rhombs	Observational, gas diffusion method, plasma polymerization used to make gradient surface, pH not specified	39
C_{11}-SH surface assembled monolayer	Calcite	Quantitative, direct mixing, flow-through method<br>γ = 91 mJ m^{-2}	40
C_{16}-SH surface assembled monolayer	Calcite	γ = 86 mJ m^{-2}	
n-octadecanoic acid monolayer ($CH_3(CH_2)_{16}COOH$)	Calcite, (11.0) face	Observational, direct mixing, monolayers at interface of air-supersaturated solution, experiments also performed with Mg^{2+} present in which aragonite precipitated	41
n-eicosyl sulfate monolayer ($CH_3(CH_2)_{19}OSO_3H$)	Calcite, trigonal pyramidal (00.1) face		
**Natural/ synthetic mixtures or composites**			
PSS[4] and proteins from *M. californianus* (bivalve)	Calcite, 17.5% total crystallites oriented onto substrate	Observational, ammonium carbonate method, protein presence increased % calcite 3 to 17.5%,<br>Suggest sulfonate and carboxylate cooperation	16
PSS[4] and proteins from *M. californianus* (bivalve) without carboxylate	Calcite, 3-5.7% of total crystallites oriented onto substrate	Observational, ammonium carbonate method, carboxylate blocked by crosslinking	
Polymer with gradient amine content and Chondroitin sulfate	Calcite, sphere or ellipsoid, much higher density where amine content is high	Observational, gas diffusion method, plasma polymerization to make gradient surface, pH not specified	39
Chitosan and PAMPS[8]-co-PAA[2]	Vaterite, 88%<br>Calcite, 12%<br>Rough spheres, aggregates	Observational, direct mixing, suggest chain rigidity influences morphology	6
Chitosan and PAMPS[8]-co-PAA[2]	Vaterite, 88%<br>Calcite, 12%<br>Rough spheres	Observational, direct mixing, smaller, more numerous crystals as concentration of PAMPS[8]-co-PAA[2] increases, larger fraction of calcite when chitosan present	35
PVS[5] grafted chitosan	All polymorphs, various morphologies (spherical, disk, prismatic, needle)	Observational, gas diffusion method, $CaSO_4$ also present due to crosslinker releasing SO_4^{2-}	42
β-cyclodextrin-maleic anhydride, PSS[4] terpolymer and chitosan	Aggregates and spherical granules, up to 96% inhibition	Observational, scale inhibition test, % inhibition increased as % monomer conversion to PSS[4] increased	43
Cellulose-based woven material and PAMPS[8]	Calcite composite layer, crystallites 576 Å	Observational, ammonium carbonate method, control produced thin calcite layer,	44

		while some larger crystals were present in composite layer(s)	
Cellulose-based woven material and PAMPS[8]-co-PAA[2]	Calcite composite layer, crystallites 163 Å		
Cellulose-based woven material with chitosan and PAMPS[8]	Calcite composite layer, crystallites 979 Å		
Cellulose-based woven material with chitosan and PAMPS[8]-co-PAA[2]	Calcite composite layer, crystallites 984 Å		
Cellulose-based woven material with poly(allylamine hydrochloride) and PAMPS[8]	Calcite composite layer, crystallites 401 Å		
Cellulose-based woven material with poly(allylamine hydrochloride and PAMPS[8]-co-PAA[2]	Calcite composite layer, crystallites 939 Å		

[1]Amorphous calcium carbonate; [2]Polyacryllic acid; [3]Octocania proteoglycan from rat with chondroitin sulfate linkages; [4]Polystyrene sulfonate; [5]polyvinyl sulfate; [6]Cetyltrimethyl ammonium; [7]Poly(diallyldimethylammonium chloride); [8]Poly(2-acrylamido-2-methyl-1-propanesulfonic acid); [9]Polymethyl siloxane

Table S2: Synthesis conditions for each sulfated chitosan material used in this study.

***O*-sulfation**						
**Sample**	**Chitosan (g)**	**HSO_3Cl (mL)**	**HCOOH (mL)**	**DMF (mL)**	**Temp. (°C)**	**Reaction time (h)**
OSC 0.23	0.501	2	15	95	50	3
OSC 0.42	1.020	7	20	200	50	3
OSC 0.77	0.504	5	20	125	50	3
***N*-sulfation**						
**Sample**	**Chitosan (g)**	**Me_3N-SO_3 (g)**	**Na_2CO_3 (g)**	**H_2O (mL)**	**Temp. (°C)**	**Reaction time (h)**
NSC 0.13 a	0.3506	0.8753	0.4547	60	50	4
NSC 0.13 b	0.815	2.0495	1.2015	150	50	3
NSC 0.28	0.5099	1.3597	0.8683	50	60	12
NSC 0.47	0.5080	1.3690	0.8680	50	60	24

Table S3: Solutions for nucleation experiments were prepared based upon the concentrations required to achieve a desired supersaturation and a near-one activity ratio upon mixing. The compositions targeted a reaction pH of 9.7 but actual experimental pH values ranged from 9.7 – 10.0 upon mixing. Thus, σ varied slightly based on the pH of each mixed solution.

Desired σ upon mixing	mM $CaCl_2$ (pre-mixing)	mM $NaHCO_3$ (pre-mixing)	$a_{Ca^{2+}}:a_{CO_3^{2-}}$ upon mixing
4.67	3.00	7.50	1.00
4.87	3.50	10.00	1.01
5.04	4.00	11.00	1.03
5.26	4.80	12.50	1.05
5.43	5.40	14.00	1.03
5.66	6.50	14.25	1.02

Figure S1: The intrinsic viscosity and molecular weight of the starting chitosan material (Chitosan A) were estimated by determining the inherent viscosity (η_{inh}) for a series of concentrations at ambient temperature in pH 4.5 DI H_2O/ acetic acid.

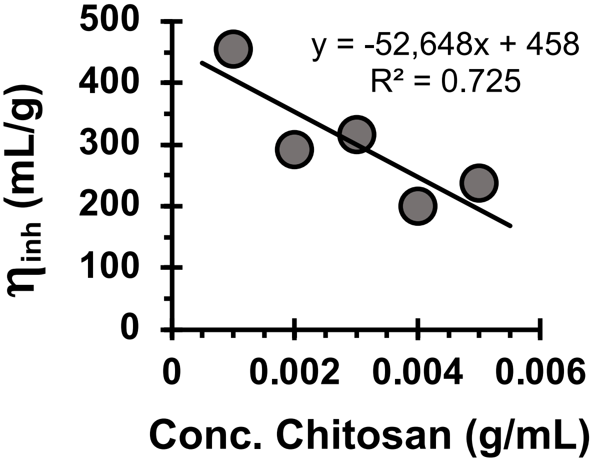

Figure S2: Aqueous SEC chromatograms for OSC 0.77 (**left**) and NSC 0.47 (**right**) collected at 50 °C in pH 3.0 DI H_2O/ acetic acid.

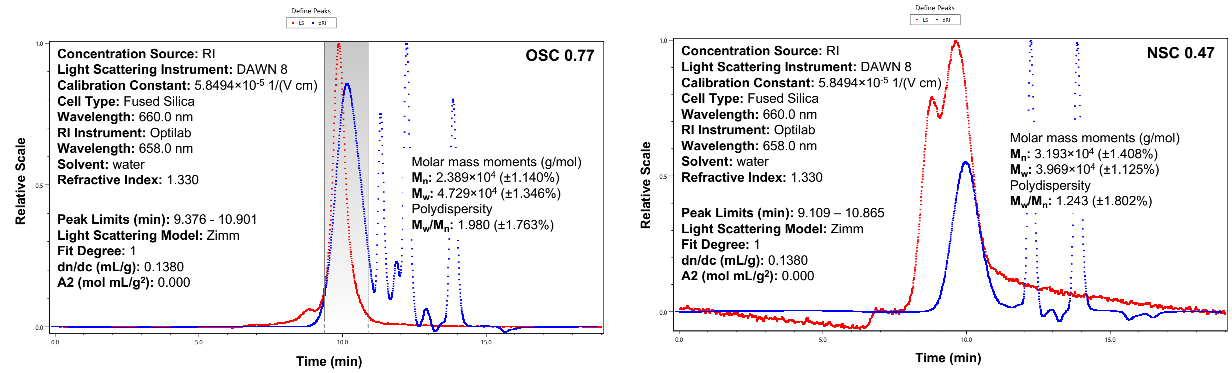

Figure S3: Example of data from NMR diffusometry measurements of the diffusion coefficient for chitosan materials. (**Left**) ^{1}H NMR spectra of chitosan over various gradient strengths collected at 25 °C on a 9.4 T Bruker Avance III. Water signal is removed. (**Right**) The intensity of the peak corresponding to the methyl protons of the acetyl group (*2.06 ppm for chitosan) over experiment number to display an exponential dependence (Mnova). The intensity (I) vs gradient strength (g) data is fit with the Stejskal-Tanner equation (using Topspin) to extract the diffusion coefficient (D) using the equation (inset) where I_0 is the signal intensity in the absence of a gradient, γ is the gyromagnetic ratio (γ_{1H} = 26.75 x 10^{7} rad T^{-1} s^{-1}), δ is the gradient pulse length (2 ms), and Δ is the diffusion time (20 – 30 ms). The average error for diffusion measurements is < 10%.

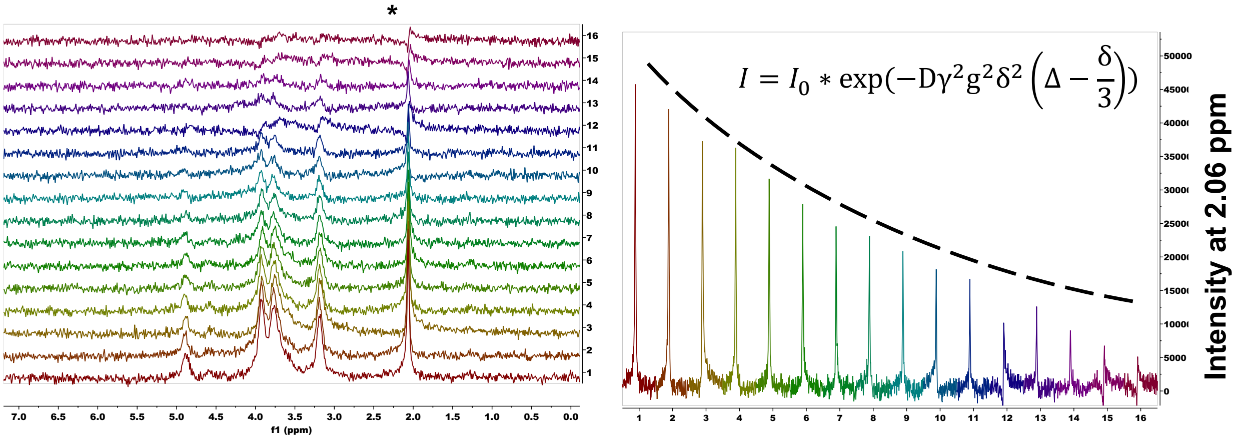

Figure S4: SEM image of a typical surface at the end of a nucleation experiment with rhombohedral calcite crystals on a relatively uniform polysaccharide substrate. The sample shown is from an experiment with *N*-sulfated chitosan surface with DS(SO_3^-) = 0.28. Magnification = 500x

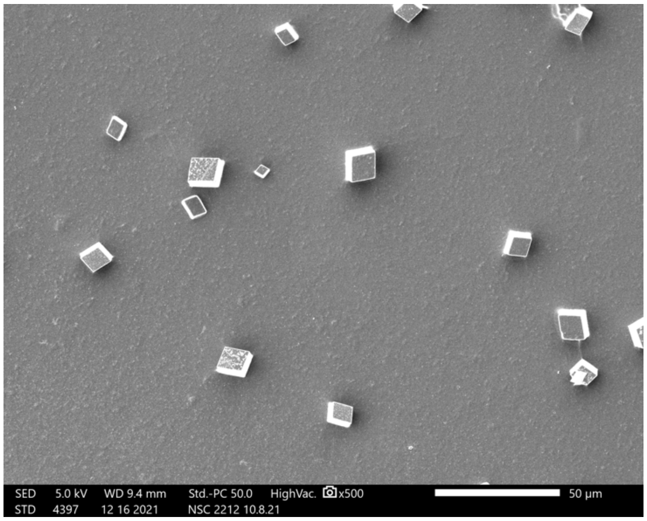

Figure S5: A representative X-ray diffractogram shows the calcite polymorph formed during nucleation onto an *N*-sulfated chitosan surface, DS(SO_3^-) = 0.28.

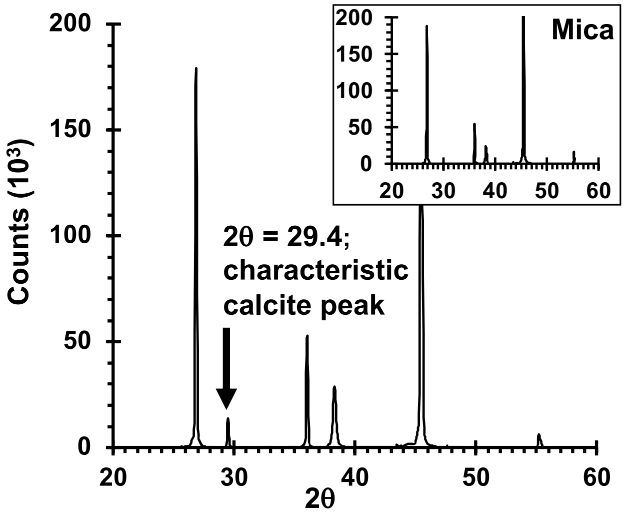

Figure S6: End-to-end distances, calculated using the CHARMM MD trajectories, show no major changes over the course of the simulation, suggesting an equilibrium conformation was adopted.

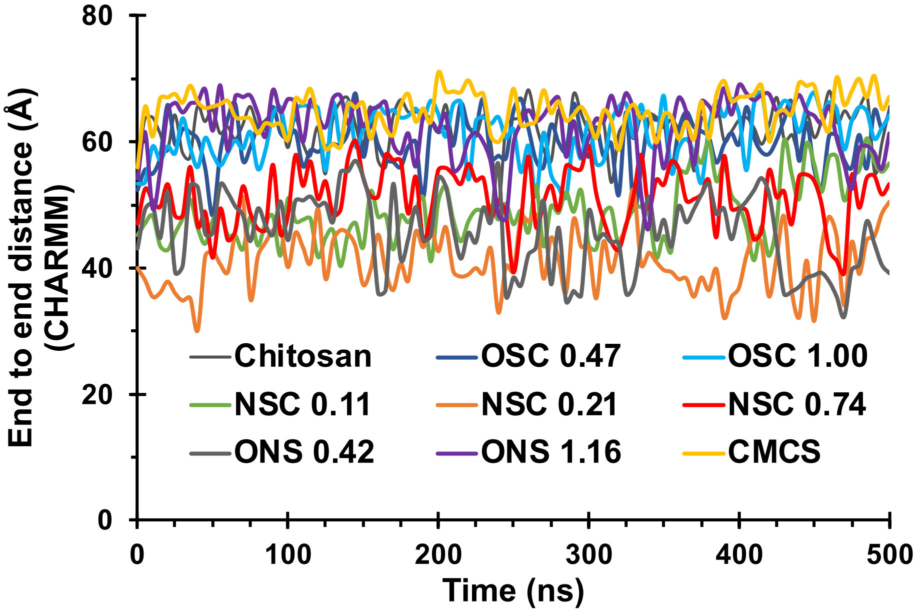

Figure S7: End-to-end distances, calculated from the AMOEBA MD trajectories, show all materials maintain an approximately constant conformation throughout the simulation. The composition ONS 0.42 exhibits a significantly lower end-to-end distance than other polymers, indicating a more intertwined/ tangled orientation.

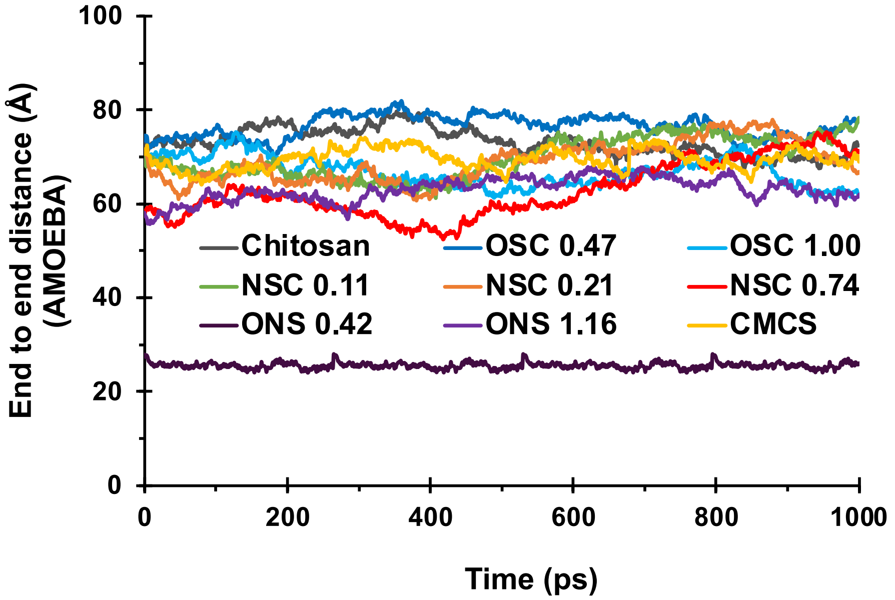

Figure S8: Nuclei vs time data for investigated materials. To obtain flux, *J*, the crystallite count is converted from number per viewing window to SI units (see statement of assumptions in manuscript).

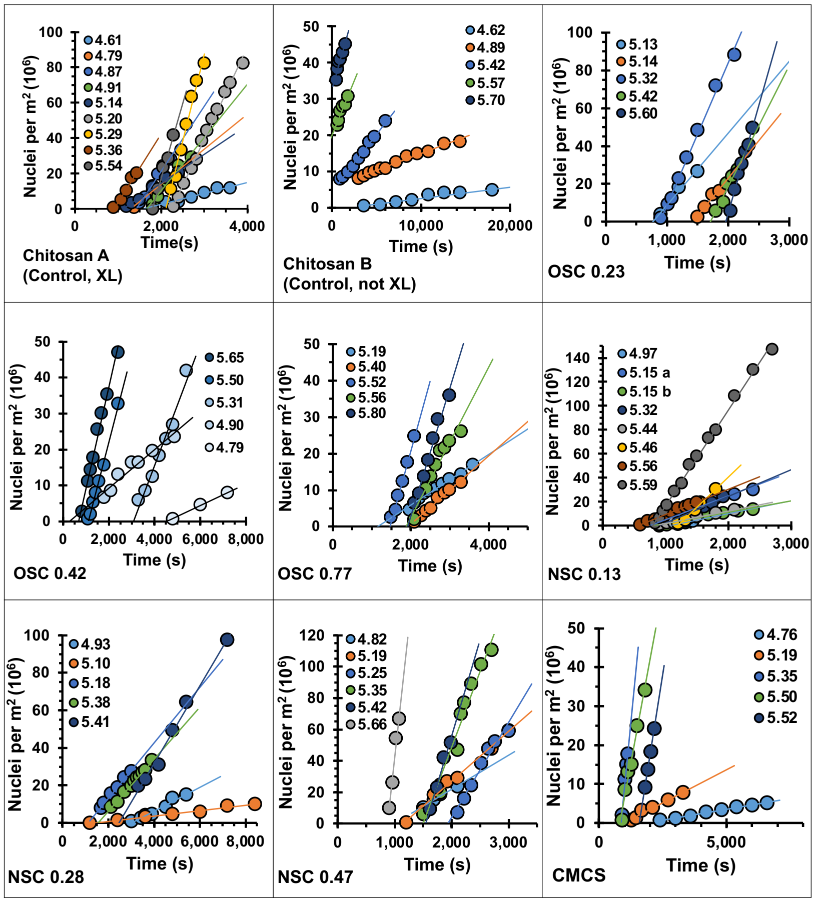

Table S4: Values of B and ln(A) for each material and the corresponding estimates of γ_{net} values that are represented in **Figures 3 - 7**.

Material	DS(Anionic group)	B ± SE[1]	ln(A) ± SE[1]	γ_{net} (mJ m^{-2})
**This study**				
Chitosan A	0	148 ± 74	16.2 ± 2.6	57
Chitosan B	0	186 ± 11	14.6 ± 0.4	59
OSC 0.23	0.23	171 ± 30	17.1 ± 1.1	57
OSC 0.42	0.42	182 ± 10	16.0 ± 0.4	58
OSC 0.77	0.77	273 ± 86	17.8 ± 2.9	66
NSC 0.13	0.13	162 ± 81	15.7 ± 2.9	56
NSC 0.28	0.28	217 ± 172	17.1 ± 6.4	62
NSC 0.47	0.47	230 ± 47	19.5 ± 1.7	63
CMCS	1.6	346 ± 99	22.1 ± 3.6	72
**Giuffre et al., *PNAS* (2014)**				
Chitosan (not XL)	0	121 ± 22	not reported	51
Hyaluronic acid	0.5	253 ± 58	not reported	65
Low G-block alginate	1	294 ± 11	not reported	72
High G-block alginate	1	390 ± 45	not reported	76
DeN- sulfated heparin	1.3	376 ± 60	not reported	75

[1]Standard error

Figure S9: (**left**) Simulations of the two *O*- and *N*-sulfated chitosan (ONS) materials also predict Ca^{2+} is solvent-separated from the sulfate groups. Ca^{2+} is concentrated in the low water density region at ≈ 5 Å with a secondary population of Ca^{2+} at ≈ 8 Å (purple). (**right**) Full S-Ca^{2+} RDF for ONS 0.42. Atoms in this system are more concentrated near the polymer due to its tangled conformation (significantly lower end-to-end distance, **Figure S7**).

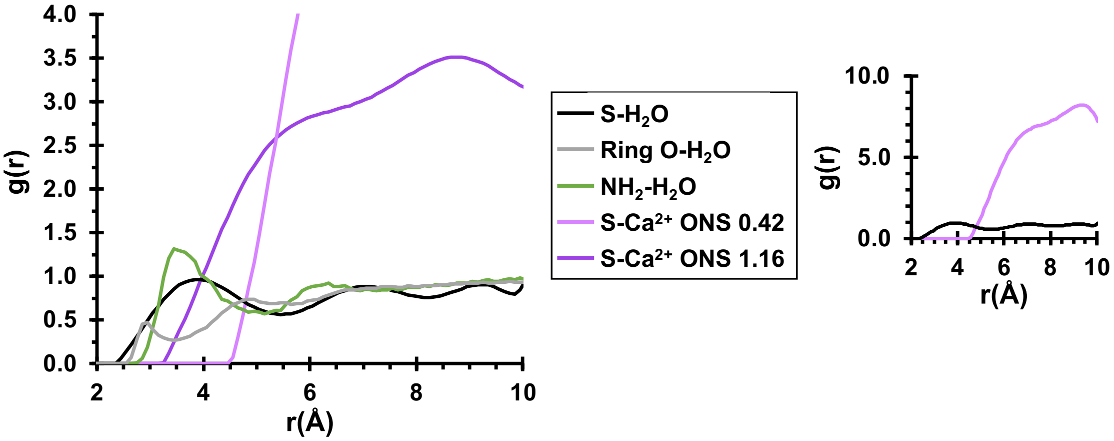

Figure S10: (left) Smallest distance between a sulfated group and Ca^{2+} is inversely correlated with $DS(SO_3^-)$ and declines to a near-constant value of ≈ 3.5 Å, independent of sulfate position. **(right)** The closest Ca^{2+}-S distances are inversely correlated with γ_{net} values determined from experiment.

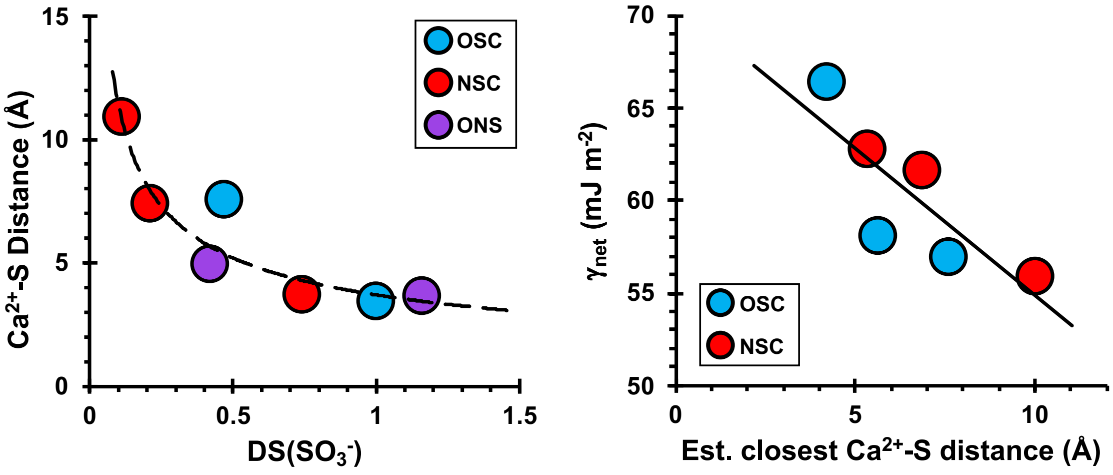

References

1. Arias, J. I.; Jure, C.; Wiff, J. P.; Fernandez, M. S.; Fuenzalida, V.; Arias, J. L. In *Effect of sulfate content of biomacromolecules on the crystallization of calcium carbonate*, Symposia on Physical Characterization of Biological Materials and Systems/Polymeric Biomaterials for Tissue Engineering/BioInspired Materials-Moving Toward Complexity, Boston, Ma, Nov 26-29; Boston, Ma, 2001; pp 243-248.
2. Guan, Y. B.; Wang, X. H.; Zhang, Q.; Sun, X. Y. In *Function of heparin in the formation biomineral: A study of biomimetic synthesis calcium carbonate*, 2nd International Conference on Chemical Engineering and Advanced Materials (CEAM 2012), Guangzhou, PEOPLES R CHINA, Jul 13-15; Guangzhou, PEOPLES R CHINA, 2012; pp 1233-1236.
3. Liu, X. R.; Zhu, B. Q.; Shao, Y. Y.; Yang, X. L., Control of morphology and structure of calcium carbonate crystals by heparin. *Chin. Sci. Bull.* **2010,** *55* (11), 1107-1111.
4. Rao, A.; Berg, J. K.; Kellermeier, M.; Gebauer, D., Sweet on biomineralization: effects of carbohydrates on the early stages of calcium carbonate crystallization. *Eur. J. Mineral.* **2014,** *26* (4), 537-552.
5. Rao, A.; Fernandez, M. S.; Colfen, H.; Arias, J. L., Distinct Effects of Avian Egg Derived Anionic Proteoglycans on the Early Stages of Calcium Carbonate Mineralization. *Cryst. Growth Des.* **2015,** *15* (5), 2052-2056.
6. Mihai, M.; Schwarz, S.; Simon, F., Nonstoichiometric Polyelectrolyte Complexes Versus Polyanions as Templates on CaCO3-Based Composite Synthesis. *Cryst. Growth Des.* **2013,** *13* (7), 3144-3153.
7. Manoli, F.; Dalas, E., Spontaneous precipitation of calcium carbonate in the presence of chondroitin sulfate. *J. Cryst. Growth* **2000,** *217* (4), 416-421.
8. Butler, M. F.; Glaser, N.; Weaver, A. C.; Kirkland, M.; Heppenstall-Butler, M., Calcium carbonate crystallization in the presence of biopolymers. *Cryst. Growth Des.* **2006,** *6* (3), 781-794.
9. Fried, R.; Mastai, Y., The effect of sulfated polysaccharides on the crystallization of calcite superstructures. *J. Cryst. Growth* **2012,** *338* (1), 147-151.
10. Hardikar, V. V.; Matijevic, E., Influence of ionic and nonionic dextrans on the formation of calcium hydroxide and calcium carbonate particles. *Colloids and Surfaces a-Physicochemical and Engineering Aspects* **2001,** *186* (1-2), 23-31.
11. Borman, A. H.; Dejong, E. W.; Huizinga, M.; Kok, D. J.; Westbroek, P.; Bosch, L., The role in CaCO3 crystallization of an acid Ca-2+-binding polysaccharide associated with coccoliths of emiliania-huxleyi. *Eur. J. Biochem.* **1982,** *129* (1), 179-183.
12. Shahlori, R.; McDougall, D. R.; Mata, J. P.; McGillivray, D. J., Effect of acid molecules on biomimetic mineralisation of calcium phosphate and carbonate within biopolymer films using small angle neutron scattering. *Physica B-Condensed Matter* **2018,** *551*, 297-304.
13. Shahlori, R.; McDougall, D. R.; Waterhouse, G. I. N.; Yao, F. H.; Mata, J. P.; Nelson, A. R. J.; McGillivray, D. J., Biomineralization of Calcium Phosphate and Calcium Carbonate within Iridescent Chitosan/Iota-Carrageenan Multilayered Films. *Langmuir* **2018,** *34* (30), 8994-9003.
14. Fernandez, M. S.; Moya, A.; Lopez, L.; Arias, J. L., Secretion pattern, ultrastructural localization and function of extracellular matrix molecules involved in eggshell formation. *Matrix Biol.* **2001,** *19* (8), 793-803.

15. Wu, T.-M.; Rodriguez, J. P.; Fink, D. J.; Carrino, D. A.; Blackwell, J.; Capalan, A. I.; Heuer, A. H., Crystallization studies on avian eggshell membranes: Implications for the molecular factors controlling eggshell formation. *Matrix Biol.* **1995,** *14* (6), 507-513.
16. Addadi, L.; Moradian, J.; Shay, E.; Maroudas, N. G.; Weiner, S., A chemical model for the cooperation of sulfates and carboxylates in calcite crystal nucleation: Relevance to biomineralization. *Proc Natl Acad Sci U S A* **1987,** *84* (9), 2732-2736.
17. Marie, B.; Luquet, G.; De Barros, J. P. P.; Guichard, N.; Morel, S.; Alcaraz, G.; Bollache, L.; Marin, F., The shell matrix of the freshwater mussel Unio pictorum (Paleoheterodonta, Unionoida). *Febs J.* **2007,** *274* (11), 2933-2945.
18. Marie, B.; Zanella-Cleon, I.; Corneillat, M.; Becchi, M.; Alcaraz, G.; Plasseraud, L.; Luquet, G.; Marin, F., Nautilin-63, a novel acidic glycoprotein from the shell nacre of Nautilus macromphalus. *Febs J.* **2011,** *278* (12), 2117-2130.
19. Fernandez, M. S.; Arias, J. I.; Neira-Carrillo, A.; Arias, J. L., Austromegabalanus psittacus barnacle shell structure and proteoglycan localization and functionality. *J. Struct. Biol.* **2015,** *191* (3), 263-271.
20. Rodriguez-Navarro, A. B.; Grenier, C.; Checa, A. G.; Jimenez-Lopez, C.; Sanchez-Sanchez, P.; Bertone, D.; Lagos, N. A., Role of the Organic Matter in the Structural Organization of Giant Barnacle Austromegabalanus Psittacus Shell from the Micro- to Nanoscale. *Cryst. Growth Des.* **2021,** *21* (1), 357-365.
21. Lu, W. F.; Zhou, D.; Freeman, J. J.; Thalmann, I.; Ornitz, D. M.; Thalmann, R., In vitro effects of recombinant otoconin 90 upon calcite crystal growth. Significance of tertiary structure. *Hear. Res.* **2010,** *268* (1-2), 172-183.
22. Smeets, P. J. M.; Cho, K. R.; Kempen, R. G. E.; Sommerdijk, N. A. J. M.; De Yoreo, J. J., Calcium carbonate nucleation driven by ion binding in a biomimetic matrix revealed by in situ electron microscopy. *Nat. Mater.* **2015,** *14* (4), 394-399.
23. Wang, T. X.; Colfen, H.; Antonietti, M., Nonclassical crystallization: Mesocrystals and morphology change of CaCO3 crystals in the presence of a polyelectrolyte additive. *J. Am. Chem. Soc.* **2005,** *127* (10), 3246-3247.
24. Wang, T. P.; Antonietti, M.; Colfen, H., Calcite mesocrystals: "Morphing" crystals by a polyelectrolyte. *Chem. Eur. J.* **2006,** *12* (22), 5722-5730.
25. Jada, A.; Verraes, A., Preparation and microelectrophoresis characterisation of calcium carbonate particles in the presence of anionic polyelectrolyte. *Colloids and Surfaces a-Physicochemical and Engineering Aspects* **2003,** *219* (1-3), 7-15Pii s0927-7757(03)00010-4.
26. Jada, A.; Verraes, A.; Aue, A.; Ducroquetz, C., Surface charges of anionic and cationic polyelectrolytes and their effects on the nucleation and the growth of CaCO3 crystals. *e-Polym.* **2009**, 136.
27. Imai, H.; Tochimoto, N.; Nishino, Y.; Takezawa, Y.; Oaki, Y., Oriented Nanocrystal Mosaic in Monodispersed CaCO₃ Microspheres with Functional Organic Molecules. *Cryst. Growth Des.* **2012,** *12* (2), 876-882.
28. Geng, X.; Liu, L.; Jiang, J.; Yu, S. H., Crystallization of CaCO3 Mesocrystals and Complex Aggregates in a Mixed Solvent Media Using Polystyrene Sulfonate as a Crystal Growth Modifier. *Cryst. Growth Des.* **2010,** *10* (8), 3448-3453.
29. Jada, A.; Pefferkorn, E., Smooth and rough spherical calcium carbonate particles. *J. Mater. Sci. Lett.* **2000,** *19* (23), 2077-2079.

30. Lei, M.; Tang, W. H.; Cao, L. Z.; Li, P. G.; Yu, J. G., Effects of poly (sodium 4-styrene-sulfonate) on morphology of calcium carbonate particles. *J. Cryst. Growth* **2006,** *294* (2), 358-366.
31. Wang, Y. S.; Moo, Y. X.; Chen, C. P.; Gunawan, P.; Xu, R., Fast precipitation of uniform $CaCO_3$ nanospheres and their transformation to hollow hydroxyapatite nanospheres. *J. Colloid Interface Sci.* **2010,** *352* (2), 393-400.
32. Mihai, M.; Schwarz, S.; Doroftei, F.; Simionescu, B. C., Calcium Carbonate/Polymers Microparticles Tuned by Complementary Polyelectrolytes as Complex Macromolecular Templates. *Cryst. Growth Des.* **2014,** *14* (11), 6073-6083.
33. Nagaraja, A. T.; Pradhan, S.; McShane, M. J., Poly (vinylsulfonic acid) assisted synthesis of aqueous solution stable vaterite calcium carbonate nanoparticles. *J. Colloid Interface Sci.* **2014,** *418*, 366-372.
34. Kawaguchi, H.; Hirai, H.; Sakai, K.; Sera, S.; Nakajima, T.; Ebisawa, Y.; Koyama, K., Crystallization of inorganic-compounds in polymer-solutions .1. Control of shape and form of calcium-carbonate. *Colloid Polym. Sci.* **1992,** *270* (12), 1176-1181.
35. Mihai, M.; Simionescu, B., Calcium carbonate and poly(2-acrylamido-2-methylpropanesulfonic acid-co-acrylic acid). a review. *Rev. Roum. Chim.* **2019,** *64*, 19-34.
36. Neira-Carrillo, A.; Pai, R. K.; Fernandez, M. S.; Carreno, E.; Quitral, P. V.; Arias, J. L., Synthesis and characterization of sulfonated polymethylsiloxane polymer as template for crystal growth of CaCO3. *Colloid Polym. Sci.* **2009,** *287* (4), 385-393.
37. Neira-Carrillo, A.; Pillai, S.; Pai, R. K., Selective control of calcium carbonate crystals morphologies using sulfonated polymer as additive. *J. Chil. Chem. Soc.* **2014,** *59* (1), 2308-2310.
38. Grassmann, O.; Löbmann, P., Morphogenetic control of calcite crystal growth in sulfonic acid based hydrogels. *Chem. Eur. J.* **2003,** *9* (6), 1310-1316.
39. Liu, X. J.; Elkhooly, T. A.; Zhang, R. R.; Feng, Q. L.; Bachhuka, A.; Vasilev, K.; Cai, Q.; Mi, S. L., Selective deposition of CaCO3 on chemical gradient surface generated by plasma polymerization and its effect on cell adhesion. *Mater. Lett.* **2017,** *186*, 90-93.
40. Hamm, L. M.; Giuffre, A. J.; Han, N.; Tao, J.; Wang, D.; De Yoreo, J. J.; Dove, P. M., Reconciling disparate views of template-directed nucleation through measurement of calcite nucleation kinetics and binding energies. *Proc. Natl. Acad. Sci. U. S. A.* **2014,** *111* (4), 1304-1309.
41. Heywood, B. R.; Mann, S., Molecular construction of oriented inorganic materials - controlled nucleation of calcite and aragonite under compressed langmuir monolayers. *Chem. Mater.* **1994,** *6* (3), 311-318.
42. Neira-Carrillo, A.; Mercade-Jaque, P.; Diaz-Dosque, M.; Tapia-Villanueva, C.; Yazdani-Pedram, M., Influence of Chitosan Grafted Poly(vinyl Sulfonic Acid) as Template on the Calcium Carbonate Crystallization. *J. Iran. Chem. Soc.* **2011,** *8* (3), 811-824.
43. Bao, Y. F.; Li, M.; Zhang, Y. Q., Research on the synthesis and scale inhibition performance of a new terpolymer scale inhibitor. *Water Sci. Technol.* **2016,** *73* (7), 1619-1627.
44. Vasiliu, A. L.; Zaharia, M. M.; Bazarghideanu, M. M.; Rosca, I.; Peptanariu, D.; Mihai, M., Hydrophobic Composites Designed by a Nonwoven Cellulose-Based Material and Polymer/CaCO3 Patterns with Biomedical Applications. *Biomacromolecules* **2022,** *23*, 89-99.
45. Giuffre, A. J.; Hamm, L. M.; Han, N.; De Yoreo, J. J.; Dove, P. M., Polysaccharide chemistry regulates kinetics of calcite nucleation through competition of interfacial energies. *Proc. Natl. Acad. Sci. U. S. A.* **2013,** *110* (23), 9261-9266.
